# Effects of Erucamide on Fiber “Softness”: Linking Single-Fiber Crystal Structure and Mechanical Properties

**DOI:** 10.1021/acsnano.4c00114

**Published:** 2024-02-09

**Authors:** Dajana Gubała, Anna Slastanova, Lauren Matthews, Luisa Islas, Patryk Wąsik, Fernando Cacho-Nerin, Dario Ferreira Sanchez, Eric Robles, Meng Chen, Wuge H. Briscoe

**Affiliations:** † School of Chemistry, University of Bristol, Cantock’s Close, Bristol BS8 1TS, U.K.; ‡ Bristol Centre for Functional Nanomaterials, HH Wills Physics Laboratory, University of Bristol, Bristol BS8 1TL, U.K.; § Diamond Light Source, Diamond House, Harwell Science and Innovation Campus, Didcot, Oxfordshire OX11 0DE, U.K.; ∥ Paul Scherrer Institut, Forschungsstrasse 111, Villigen PSI 5232, Switzerland; ⊥ Procter & Gamble Newcastle Innovation Centre, Whitley Road, Longbenton, Newcastle NE12 9TS, U.K.; # Procter & Gamble Technology (Beijing) Co., Ltd., 35 Yu’an Rd, Shunyi District, Beijing 101312, China

**Keywords:** single fiber, polypropylene fibers, erucamide, nanofocused XRD, stress-induced single-fiber crystallization, single-fiber mechanical tests, fiber softness and ductility

## Abstract

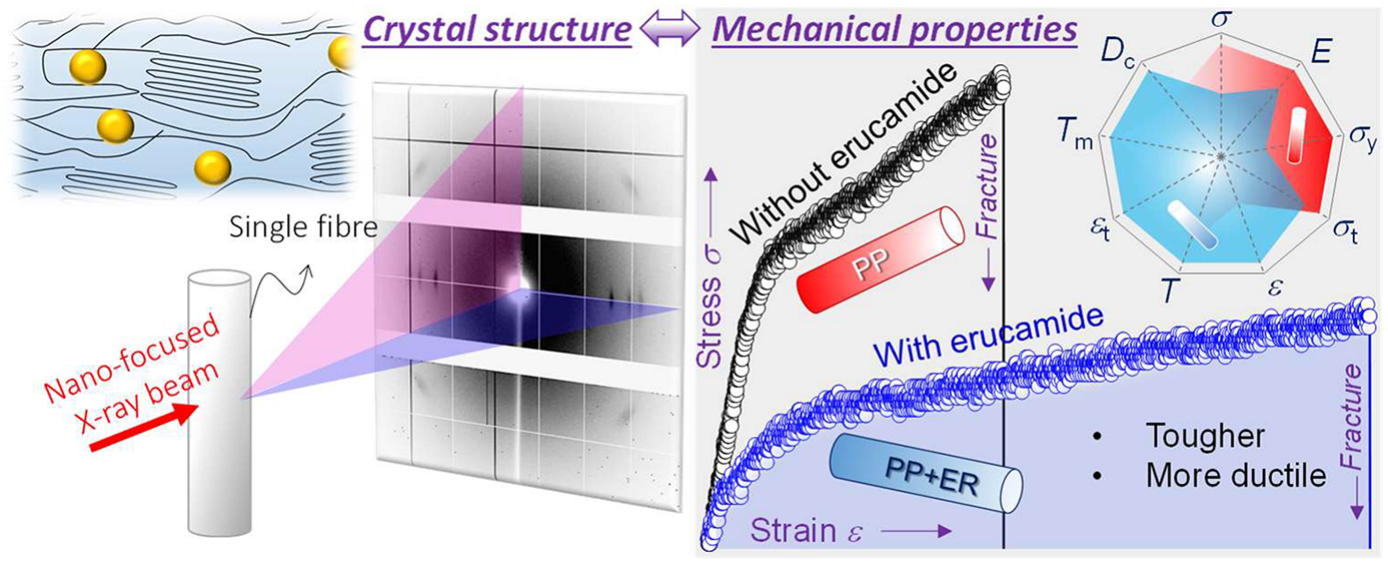

Erucamide is known to play a critical role in modifying polymer fiber surface chemistry and morphology. However, its effects on fiber crystallinity and mechanical properties remain to be understood. Here, synchrotron nanofocused X-ray Diffraction (nXRD) revealed a bimodal orientation of the constituent polymer chains aligned along the fiber axis and cross-section, respectively. Erucamide promoted crystallinity in the fiber, leading to larger and more numerous lamellae crystallites. The nXRD nanostructual characterization is complemented by single-fiber uniaxial tensile tests, which showed that erucamide significantly affected fiber mechanical properties, decreasing fiber tensile strength and stiffness but enhancing fiber toughness, fracture strain, and ductility. To correlate these single-fiber nXRD and mechanical test results, we propose that erucamide mediated slip at the interfaces between crystallites and amorphous domains during stress-induced single-fiber crystallization, also decreasing the stress arising from the shear displacement of microfibrils and deformation of the macromolecular network. Linking the single-fiber crystal structure with the single-fiber mechanical properties, these findings provide the direct evidence on a single-fiber level for the role of erucamide in enhancing fiber “softness”.

## Introduction

**Figure 1:**
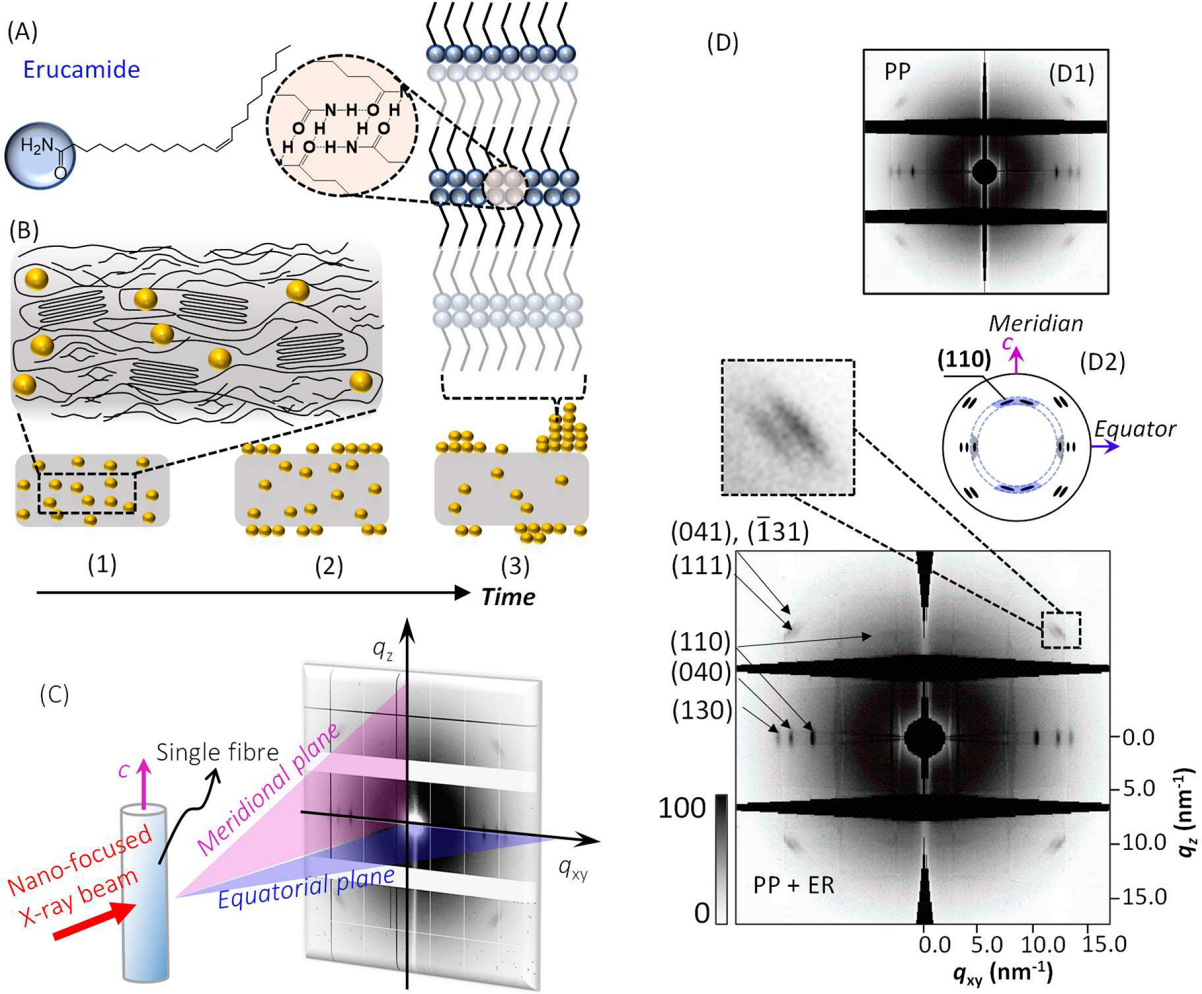
(A) Erucamide chemical structure. (B) Schematic representation of the migration (i.e., blooming) process of erucamide molecules (yellow dots) through the polymer matrix (gray): (1) erucamide dispersed the amorphous fraction surrounding the crystallites; (2) migration of erucamide to the fiber surface; and (3) formation of surface multilamellar structures, with the enlarged view showing hydrogen bonding between adjacent amide headgroups. (C) Schematic representation of the geometry of nanofocused X-ray diffraction by a vertically aligned fiber (fiber *c*-axis shown in pink), showing the meridional (or vertical) plane (*q*_*z*_) and the equatorial (or horizontal) plane (*q*_*xy*_) scattering vectors on the 2D detector plane. (D) Example XRD diffractogram of a polypropylene single fiber with erucamide (PP + ER), with the Bragg reflections assigned to isotactic α-polypropylene (compared with that for PP shown in (D1)). The enlarged view shows the off-meridional and off-equatorial Bragg reflections. (D2) The schematic representation showing (110) Bragg reflections on the fiber equator (gray) and near the meridian (blue) (110). More XRD results obtained for single PP and PP + ER fibers are shown in Supporting Information SI.05. The monoclinic crystal unit cell parameters (cf. Section SI.02) averaged from two samples per fiber type, yielded *a* = 6.67 ± 0.01 Å, *b* = 21.12 ± 0.04 Å, *c* = 6.49 ± 0.03 Å, α = γ = 90° and β = 98.57 ± 0.01° for PP and *a* = 6.67 ± 0.01 Å, *b* = 21.08 ± 0.08 Å, *c* = 6.48 ± 0.01 Å, α = γ = 90° and β = 98.35 ± 0.19° for PP + ER. The unit cell volume (eq S6) and the crystalline density (eq S7) yielded very similar values of 906.2 ± 6.0 Å^3^ and 0.924 ± 0.06 g·cm^–3^ for PP, and 901.1 ± 4.7 Å^3^ and 0.929 ± 0.05 g·cm^–3^ for PP + ER, respectively. These values are in agreement with those reported for the α-form of isotactic polypropylene.^43,44^

Polymer fibers are found in a vast array of applications, ranging from textiles to healthcare and personal care products.^1−4^ To attain desired fiber surface characteristics, small additive molecules (i.e., long-chain fatty acid amides often termed *slip additives*) are applied to the host polymer prior to fiber extrusion, which undergo subsequent migration through the amorphous fraction to the fiber surface (Figure 1).^5^ The surface additive concentration typically yields at ∼40% of the original bulk loading, leaving an appreciable fraction of the additives suspended in the amorphous regions of the polymer matrix.^6−8^

Erucamide (13-*cis*-docosenamide, C_22_H_43_NO; cf. Figure 1A) has been widely used as a slip additive, effectively altering polymer surface morphology and nanomechanical properties (i.e., surface roughness, adhesion, and friction).^1,8−15^ As an amphiphilic molecule, erucamide comprises an unsaturated hydrophobic chain and a hydrophilic primary amide headgroup and its distribution in the polymer is highly dependent on its concentration and the chemistry of the host polymer matrix,^16,17^ as well as fiber processing parameters.^17−21^ For instance, Quijada Garrido et al.,^22^ using wide-angle X-ray scattering (WAXS) and differential scanning calorimetry (DSC), revealed erucamide crystalline microdomains and globules of 0.3–10 μm in size situated partially within or around the amorphous region and partially on the surface of isotactic polypropylene (PP) spherulitic films (cf. Figure 1B).

It is more challenging to evaluate the effect of erucamide on *polymer fibers* due to the complex fiber geometry. We have previously studied single fibers using atomic force microscopy (AFM) and a spray-on wettability test method.^1^ Erucamide was found to form patchy multilayers, with its hydrophobic tails oriented outward (Figure 1B) on the surface of single PP fibers,^1^ and hydrophilic^15^ and hydrophobic^14^ substrates. The results thus established the link between the erucamide structure and its efficacy to tune fiber surface wettability and adhesion.

The mechanical properties of polymer fibers are critical to their processing and performance and underpin fiber “softness” which is a key consumer satisfaction criterion in many fiber products. With human tactile perception sensitive to surface features on a nanoscale,^23^ erucamide has the potential for optimization of consumer perception related to softness of spunbond nonwovens in healthcare products (e.g., surgical masks and protective gowns) and personal care products (e.g., nappies and sanitary pads).^24,25^ Furthermore, the presence of erucamide is thought to moderate fiber to fiber friction, limiting the occurrence of fiber breakage during the manufacturing process.

Previous studies have focused on polymer films or the fiber surface effects (i.e., friction, surface roughness, morphology, and adhesion) of erucamide.^1,8−13,18,22,26^ Limited suitable experimental set-ups have been reported^27^ to probe single fibers due to fiber’s small diameter (∼10 μm), complex geometry, and low tensile strength (∼10–100 MPa).^28^

X-ray diffraction (XRD) has been used to characterize the crystal structure of synthetic and natural fibers/fabrics.^29−40^ Our previous XRD study^1^ on bundles of randomly oriented PP fibers did not observe appreciable changes in the fiber crystal structure in the presence of 1.5 wt % (i.e., 15000 ppm) erucamide. The X-ray beam size (320 μm × 250 μm) in that study significantly exceeded the fiber diameter (*d*_f_ < 15 μm) and the obtained lattice spacing was thus averaged over the crystallite fraction of multiple fibers of different orientations.

Despite these previous studies, there is a lack of understanding of the effects of erucamide on the local crystal structure of additive-loaded polymer fibers and how such crystal structure is correlated with fiber elastic/mechanical properties, highly relevant to fiber industrial applications. Here, we investigate the effects of erucamide on the crystal structure of a single fiber using nanofocused X-ray Diffraction (nXRD). The results are correlated with the single-fiber mechanical properties. These observations are discussed in the context of the role of erucamide in stress-induced crystallization of the melt-spun polypropylene fiber as well as its plastic deformation under the tensile stress. The fundamental knowledge of the effect of erucamide, linking the single-fiber crystal structure and single-fiber mechanical properties, is relevant to our understanding of the molecular origin of fiber “softness”, important to tuning fiber properties with erucamide as an additive widespread in industrial applications.

## Results and Discussion

### Single Fiber Crystal Structure

We performed synchrotron nXRD (beam size 60 nm) measurements on single fibers, by scanning different sections of the fiber along its length and cross-section (details for the experimental setup, procedure, and analyses in Section SI.01). In general, the nXRD scattering intensity varied slightly along the fiber length and cross-section, indicative of localized degree of crystallinity, while the crystal structural parameters remained consistent. Figure 1 shows representative 2D diffractograms integrated along the fiber cross-section (i.e., sum of the diffraction signals in region S1 in Figure S2B) for a single polypropylene fiber (PP; inset figure D(1)) and polypropylene fiber with 1.5 wt % erucamide (PP + ER; main figure), respectively. Table 1 lists the fitted crystal structural parameters for polypropylene (PP) and polypropylene fiber with erucamide (PP + ER) averaged at the four sections (S_1_–S_4_; cf. Figure S2) over two fibers each fiber type, with the full data set listed in Table S1 and S2 in Supplementary SI.04). All the Bragg reflections could be assigned to isotactic α-polypropylene, which corroborates with our previous XRD findings on *fiber bundles*.^1^

**Table 1:** Fitted Crystal Structural Parameters for Polypropylene (PP) and Polypropylene Fibers with Erucamide (PP + ER) Averaged at the Four Sections (S_1_–S_4_; cf. Figure S2) over Two Fibers Each Fiber Type: Average Peak Position *q*, Crystal Planes (*hkl*) and *d*-Spacing (Å), and the (Minimum) Crystallite Domain Size (*L*_a_) in the Direction Perpendicular to the (*hkl*) Plane^a^

fiber	average *q* (nm^–1^)	*hkl*	average *d*-spacing (Å)	*L*_a_ (Å)
**PP**	9.98	110 eq.	6.30 ± 0.01	175 ± 19
	9.98	110 m.	6.30 ± 0.01	105 ± 1
	11.91	040	5.28 ± 0.01	167 ± 28
	13.08	130	4.80 ± 0.01	109 ± 26
	14.92	111	4.21 ± 0.01	109 ± 50
	15.42	041/1̅31	4.08 ± 0.01	104 ± 31
	17.78	060	3.53 ± 0.01	44 ± 31
**PP + ER**	9.97	110 eq.	6.30 ± 0.01	244 ± 8
	9.97	110 m.	6.30 ± 0.01	103 ± 1
	11.93	040	5.27 ± 0.02	213 ± 29
	13.10	130	4.80 ± 0.01	151 ± 37
	14.94	111	4.21 ± 0.01	144 ± 48
	15.45	041/1̅31	4.07 ± 0.01	144 ± 34
	17.75	060	3.54 ± 0.01	84 ± 27

a The errors for the *d*-spacing were calculated via the error propagation method based on the uncertainty of the peak position, also considering the angular resolution of the instrument, which is smaller than 0.1 Å. The errors calculated as the standard deviation between the 4 sections of two fibers per sample batch yielded less than 0.06 nm^–1^ for the peak position *q*. The fitted structural parameters corresponding to the equatorial (parent lamellae) and meridional (daughter lamellae) contributions to (110) reflection are denoted as 110 eq. and 110 m., respectively. The errors for *L*_a_ are calculated via error propagation using the uncertainties in FWHM (Δ*q*). The full dataset is tabulated in Table S1 and Table S2 in SI Section SI.04.

**Figure 2:**
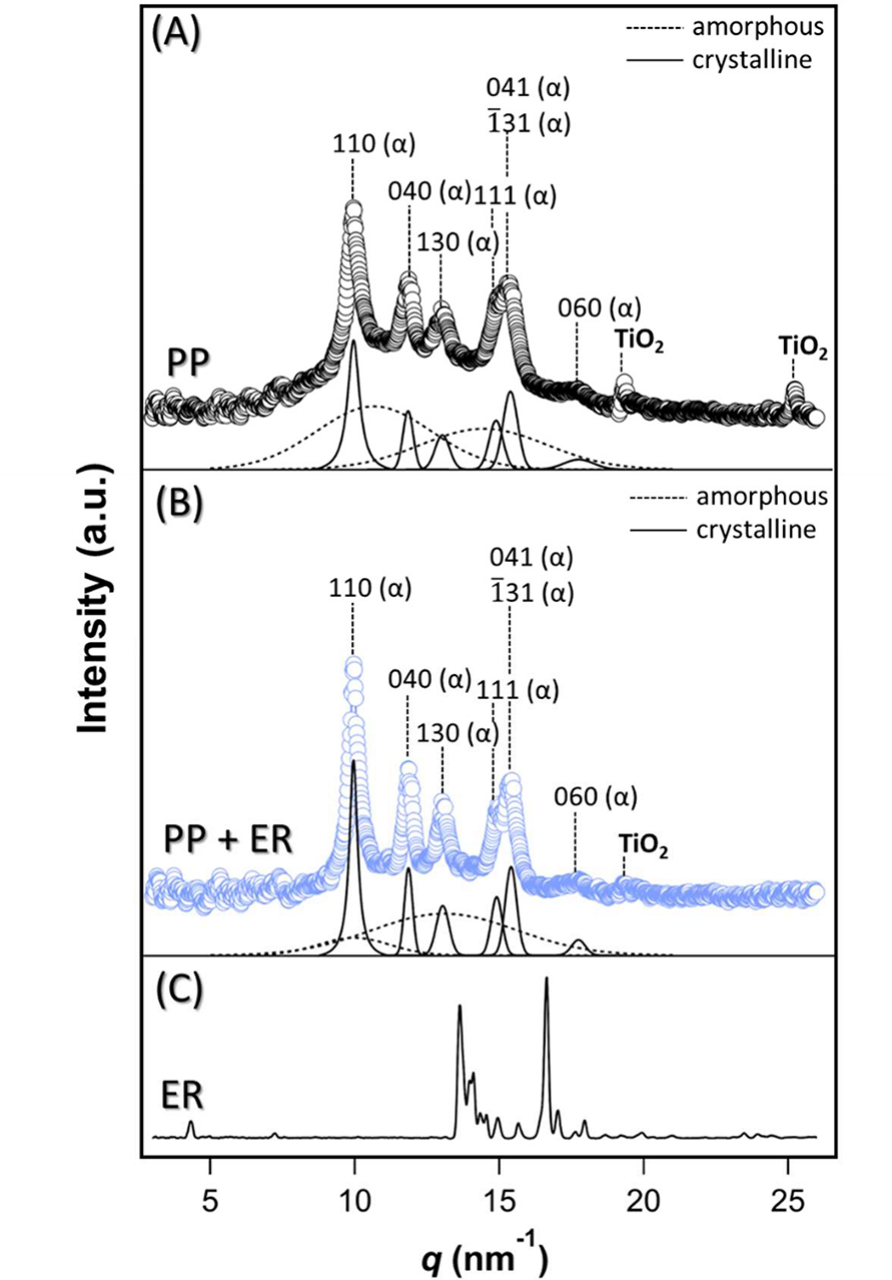
Example XRD curves for (A) a polypropylene fiber (PP), (B) a polypropylene fiber with erucamide (PP + ER), and (C) the erucamide control sample, with the peaks labeled with the assigned (*hkl*) indices. The dashed lines show the two Gaussian fits for the amorphous halo (i.e., the amorphous fraction) and the solid lines show the Gaussian and Voigt fits for the Bragg peaks (i.e., the crystalline fraction). The fitted structural parameters (i.e., the peak position *q* and the lamellar *d*-spacing) obtained at the four scanned sections along the fiber length are listed in Table S1. The presence of TiO_2_ particles in the single fiber is also confirmed by the peaks at *q* = 19.34 nm^–1^ (*d* = 3.25 Å, (101)) and *q* = 25.21 nm^–1^ (*d* = 2.49 Å, (004)),^58,59^ added as an antimicrobial, UV-protective, and whitening agent. It has been previously shown that TiO_2_ particles have a negligible effect on the polypropylene crystal structure at loadings exceeding that in the fibers studied here.^58,60,61^

Figure 2 shows representative integrated 1D plots from the 2D fiber diffractograms, along with the erucamide crystal control. Fitting to the 1D plots reveals amorphous halos and Bragg peaks, consistent with a semicrystalline structure characteristic of polymeric materials.^41^ The structural parameters (i.e., peak position, *d*-spacing, and the monoclinic crystal unit cell parameters) for PP + ER and PP corresponded closely to isotactic α-polypropylene, pointing to the polymer crystal structure being unaltered by erucamide (Table 1; see also Table S1, Section SI.04). The unit cell parameters, specifically the length along the *c*-axis (*c* = 6.5 Å), corresponding to the chain segment composed of three PP monomer units (C_3_H_6_) arranged in a 3-fold helical geometry.^42^

The absence of the erucamide Bragg peaks could be attributed to its relatively small concentration in the fiber, in agreement with XRD results on erucamide-loaded polypropylene films^22^ and fiber bundles.^1^ However, as we discuss below, the amorphous fraction constituted a larger part of the semicrystalline polymer in PP than PP + ER, pointing to the effect of erucamide on promoting fiber crystallinity.

The anisotropic 2D XRD patterns of the fiber samples (Figure 1D) are indicative of certain preferential orientations of the crystalline domains with respect to the fiber *c*-axis.^45,46^ The three low-*q* equatorial Bragg reflections are characteristic for isotactic α-polypropylene *d*-spacing (cf. Table 1) *d* = 6.30 Å, *d* = 5.28 Å (or *d* = 5.27 Å for PP + ER), and *d* = 4.80 Å, corresponding to the (110), (040), and (130) planes oriented normal to the surface of the polypropylene crystalline lamellae (001), composed of polymer chains oriented parallel to the meridian (i.e., *c*-axis).^43,47−50^ The equatorial reflections indicate a regular structural repeat in the direction along the direction of the fiber radius.

For both PP and PP + ER fibers, broad off-equatorial and off-meridional (111) and (041)/(1̅31) Bragg reflections (Figure 1D), corresponding to *d* = 4.21 Å and *d* = 4.08 Å (*d* = 4.07 Å for PP + ER)^51,52^ (cf. Table 1), originate from the helical conformation of the polypropylene chains, where (041) and (1̅31) could not be distinguished.^42,44,53^ For PP + ER, these reflections appeared as a pair of intense diffraction maxima (Figure 1D), whereas for PP one can observe a less intense, single, broad reflection (Figure 1D; also Figure S4 and Figure S5 in SI.05). The higher peak intensity from PP + ER points to a higher crystalline fraction within the polymer matrix.^22,54−57^

**Figure 3:**
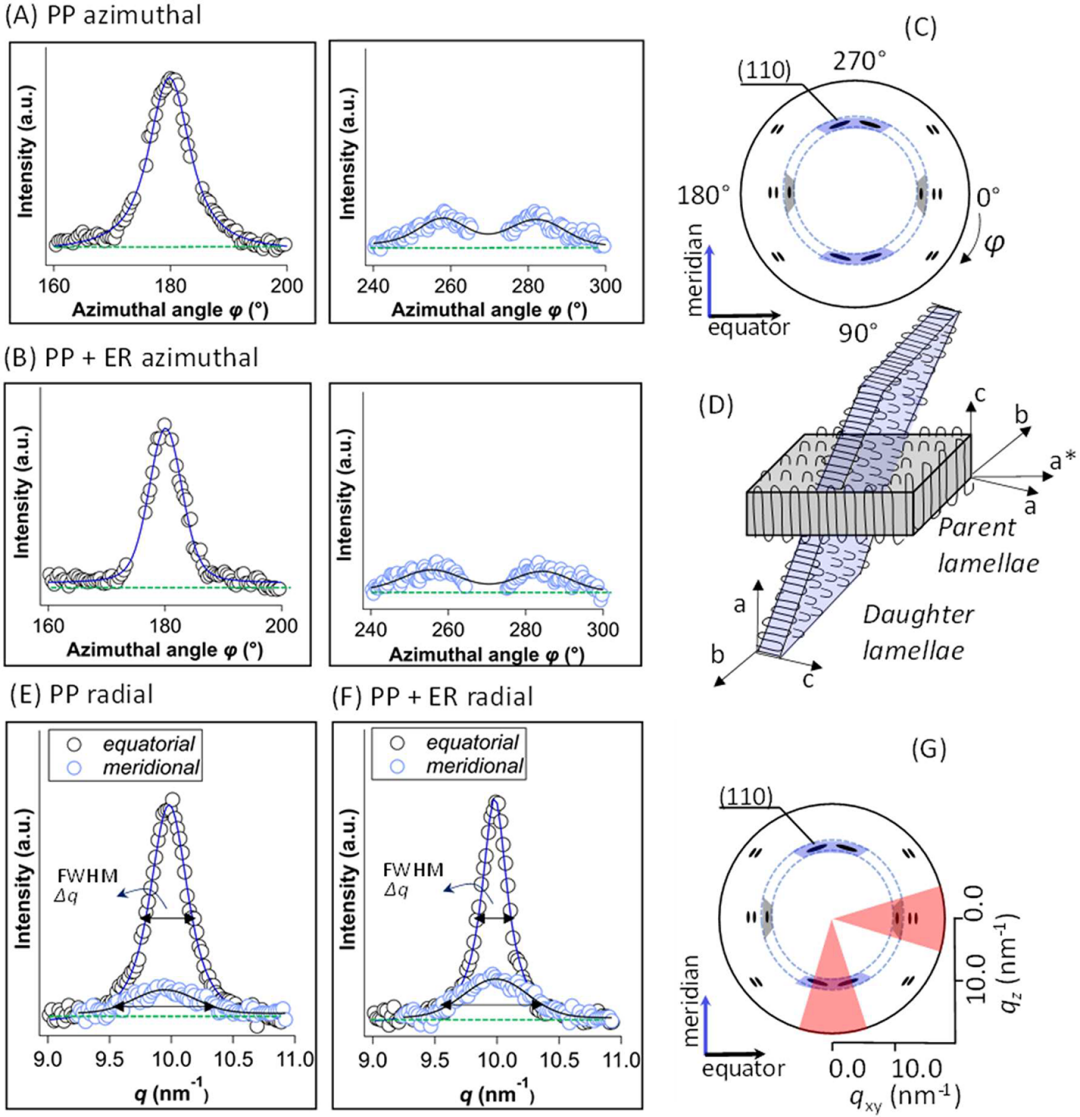
Representative azimuthal intensity distributions of equatorial (φ = 180° ± 30°) and near-meridional (φ = 270° ± 30°) (110) Bragg reflections (in the *q* range of 9–11 nm^–1^) for (A) a polypropylene fiber (PP) and (B) a polypropylene fiber with erucamide (PP + ER). (C) Schematic showing the azimuthal region corresponding to equatorial reflections in gray and the azimuthal region corresponding to near-meridional reflections in blue. (D) Schematic of the bimodal orientation of the daughter lamellae, corresponding to near-meridional reflections, growing from the parent lamellae, corresponding to equatorial reflections. *a*, *b*, and *c* represent the PP monoclinic unit cell axes and a* is the component of *a*-axis oriented perpendicular to *c* and *b* axes, and represents the growth direction of the parent lamellae. The *a*-axis and *c*-axis of the parent lamellae correspond to the *c*-axis and *a*-axis of the daughter lamellae, respectively. Representative radial intensity distributions of equatorial and near-meridional (110) Bragg reflections for (E) a PP and (F) a PP + ER fiber. (G) Schematic 2D XRD pattern with the radial region highlighted. The results from more fibers replicates can be found in Supporting Information SI.06 and SI.06.

The azimuthal intensity distributions of the (110) reflection (in the *q* range 9–11 nm^–1^; cf. Figure 1D) for both PP and PP + ER are shown in Figure 3A and 3B. The simultaneous appearance of the equatorial and near-meridional components points to the *bimodal crystalline orientation*, with *daughter* or secondary lamellae growing epitaxially atop *parent* or primary lamellae oriented along the fiber *c*-axis as shown schematically in Figure 3D.^62−67^ The bimodal orientation originates from the deviation from the alternating sequence of right-handed and left-handed helices characteristic for the isotactic α-polypropylene chains in its monoclinic unit cell.^62−64,67−70^ The synchrotron nXRD here was effective for identification of this particular structural feature in a single fiber, which is otherwise challenging to detect due to the relatively small number and size of the crystallites within the semicrystalline matrix.

Analysis of Bragg peaks using the Scherrer equation (Section SI.08; Figure S8—showing *L*_a_ and *m* from different Bragg peaks)^71−76^ yielded an estimate of the crystallite size *L*_a_∼ 100–250 Å for both PP and PP + ER single fibers (cf. Table 1; also Table S2), a value comparable to that typically reported for the thickness of the polymer microfibrils.^77^ In general, the fibers with erucamide exhibited a larger crystallite domain size. Small crystallite size variations could be observed between the fibers studied (Fiber 1 and Fiber 2), as well as at the four scanned sections (S_1_–S_4_) along the fiber length (Figure S9 and Figure S10). The radial intensity distribution of the (110) Bragg reflection (Figure 3E–G) allowed us to distinguish between the size of the parent and daughter lamellae (Figure 3 and Figure S11; fitting results in Table S2, SI.04). For both PP and PP + ER single fibers, the parent lamellae were more prevalent, i.e., a larger number of polypropylene chains oriented along the fiber length than those oriented radially. The average size of the parent lamellae was approximately twice that of the daughter lamellae (cf. Table 1; Table S2), with an average parent lamellae crystal size *L*_a_ ∼ 244 ± 8 Å for PP + ER and *L*_a_ ∼ 175 ± 19 Å for PP, respectively. This corroborates with a previous study of the crystal orientations in the PP fiber during melt spinning^67^ which reported that the daughter lamellae were smaller in size with more imperfections.

Erucamide had a negligible effect on the size of the daughter lamellae, with *L*_a_ ∼ 103 ± 1 Å for PP + ER and *L*_a_ ∼ 105 ± 1 Å for PP, respectively (cf. Table 1). However, it promoted the prevalence of the daughter lamellae, with the average relative content of the daughter to the parent lamellae across four studied sections (S_1_–S_4_) along the fiber length valuing at 16 ± 1% (daughter) to 84 ± 1% (parent) for PP + ER and 11 ± 1% (daughter) to 89 ± 1% (parent) for PP (Figure S8; Table S3).

The fine scan step-size (100 nm) of the nanofocused beam allowed the comparison at the three regions across the fiber width (cf. Figure S2 experimental setup): close to the fiber edges (A_1_, A_3_) and at the fiber core (A_2_) (cf. Figure S12, S13 and Table S5, SI.10). For PP single fibers, the crystallite size *L*_a_ was slightly larger for the fiber core (A_2_) than fiber edges (A_1_ and A_3_). This may have resulted from the stress-induced crystallization of the molten core during fiber extrusion and the cooling time difference across the fiber cross-section, with the fiber edge cooling faster than the fiber core, resulting in the orientation differences and more crystalline core compared to the edge.^84^ In the case of PP + ER, the crystallite size *L*_a_ was similar in A_1_–A_3_. We rationalize this observation as follows. Due to the incompatibility of the amphiphilic erucamide molecules with hydrophobic polyolefin, we can expect erucamide to migrate toward fiber surface upon cooling and thus enhance fiber crystallinity more prominently in the region closer to fiber edge, compensating for the crystallinity differences between fiber center and edge, observed in the PP fiber.

### Single Fiber Crystallinity

**Figure 4:**
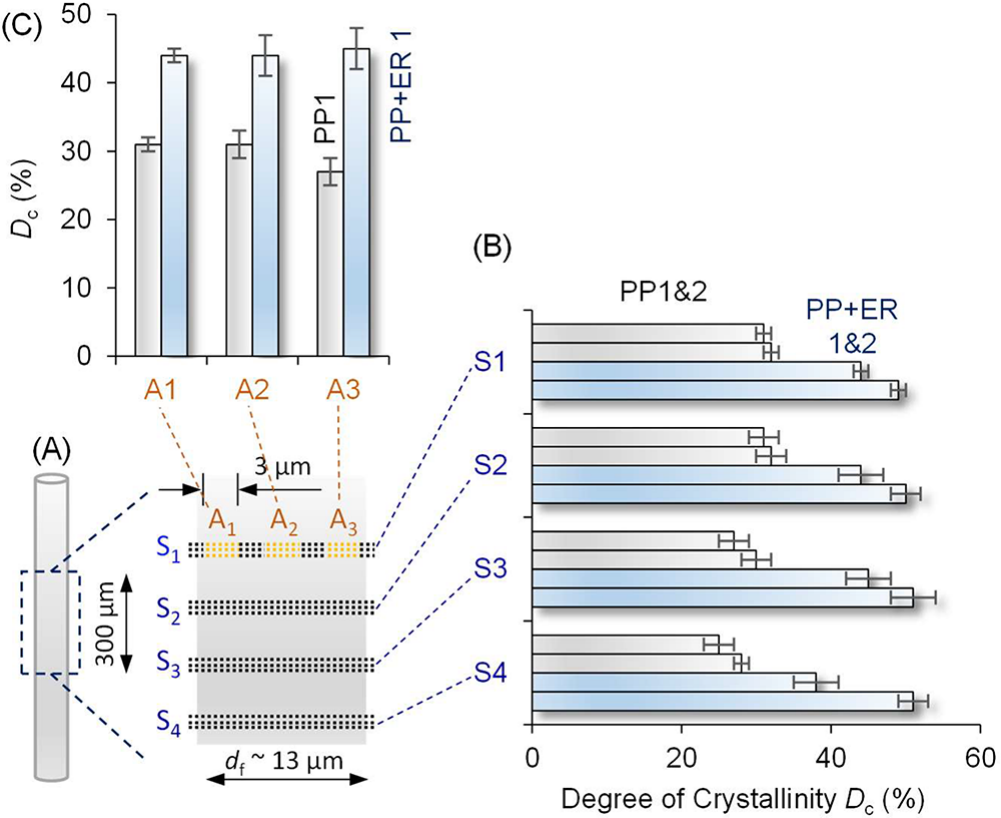
(A) Schematic showing four sections (S_1_–S_4_) scanned along 300 μm of fiber length, with 3 scans in each section 1 μm apart, with a 100 nm step size in S_1_ and a 500 nm step size in S_2_–S_4_. The crystal structure along fiber width was investigated at three areas (A_1_–A_3_) marked in orange in S_1_. (B) Degree of crystallinity (*D*_c_) for polypropylene (PP) and polypropylene with erucamide (PP + ER), two samples per fiber type, across S_1_–S_4_ (cf. Table S4 in SI.09). (C) *D*_c_ for two fibers (PP and PP + ER) at A_1_ (left edge), A_2_ (core), A_3_ (right edge) along S_1_ across the fiber width (cf. Table S5 in SI.10).

The values for the degree of crystallinity, *D*_c_, for the four sections (S_1_–S_4_) along the fiber length and for the three regions across the fiber width (A_1_–A_3_) for PP and PP + ER are shown in Figure 4 and listed in Table 2 (see also Table S4 and Table S5), with the decomposed crystalline peaks and amorphous halos shown in Figure S9, S10, S12, and S13 (SI.09 and SI.10). For all the fibers studied, the amorphous fraction comprised a substantial part of the semicrystalline polymer. Along the length of the PP single fibers, *D*_c_ = 30 ± 2% (Fiber 1) and *D*_c_ = 31 ± 1% (Fiber 2), values that are significantly lower than the erucamide-loaded PP + ER samples for which *D*_c_ = 43 ± 2% (Fiber 1) and *D*_c_ = 50 ± 2% (Fiber 2) (cf. Figure 4B). That is, despite erucamide having no observable effect on the α-polypropylene unit cell, it promoted fiber crystallinity. The results are in agreement with thermal analysis of the samples attained via differential scanning calorimetry (DSC; cf. Figure S15 in SI.11), which confirmed that the melting temperature of more crystalline PP + ER (*T*_m_ = 160.7 ± 0.1 °C) was higher than melting temperature of PP (*T*_m_ = 149.1 ± 0.2 °C).

**Table 2:** Mechanical Parameters of Polypropylene (PP) and (PP + ER) Fibers: Engineering Tensile Strength (σ), Engineering Elongation at Break (ε), True Tensile Strength (σ_t_), True Elongation at Break (ε_t_), Young’s Modulus (*E*), Yield Strength (σ_y_), and Toughness (*T*)^a^

sample	σ (MPa)	ε (%)	σ_t_ (MPa)	ε_t_ (%)	*E* (GPa)	σ_y_ (MPa)	*T* (Jm^–3^)	*T*_m_ (°C)	*D*_c_ (%)
**PP**	344 ± 25	71 ± 6	586 ± 43	53 ± 3	1.5 ± 0.3	201 ± 22	153 ± 16	149.1 ± 0.2	29.6 ± 2.4
**PP + ER**	192 ± 6	162 ± 6	499 ± 20	96 ± 2	1.2 ± 0.2	80 ± 6	188 ± 8	160.7 ± 0.1	45.4 ± 4.0

a The errors were calculated as standard deviations from 10 fibers. Also listed are the DSC melting temperature *T*_m_ (cf. SI.11) and the degree of crystallinity (*D*_c_) for PP and PP + ER (averaged from 11 measurements of each fibre type).

For PP, *D*_c_ was slightly smaller at the fiber edges (regions A_1_ and A_3_; *D*_c_ = 28 ± 3%) than the fiber core (region A_2_; *D*_c_ = 34 ± 2%) for Fiber 1, and the values for Fiber 2 are *D*_c_ = 29 ± 3% (edges) and *D*_c_ = 33 ± 2% (core) (cf. Figure 4C; Table S5). This corroborates with the larger crystallite size in the PP fiber core discussed above. For PP + ER, *D*_c_ appeared more consistent for all three regions (A_1_–A_3_) across the fiber width, with *D*_c_ = 44 ± 4% (Fiber 1) and 50 ± 4% (Fiber 2), consistent with the uniform crystallite size across the fiber width.

In general, small variations in the crystallite size and *D*_c_ values observed between the two fibers of the same type (Fiber 1 and Fiber 2), as well as along the fiber length and width, may have resulted from the variabilities in the processing conditions such as temperature and pressure gradients during injection molding, fiber extrusion and drawing, as well as solidification time, all of which could contribute to the final crystal structure of the polymer.^36^ Furthermore, the local degree of crystallinity of PP + ER could also have been affected by an uneven distribution of the additive molecules which is susceptible to thermodynamic (e.g., temperature, cooling rate, and aging time) and kinetic parameters (e.g., molecular structures of polymer and additive).^7,16,21,85−90^

### Single Fiber Mechanical Properties

**Figure 5:**
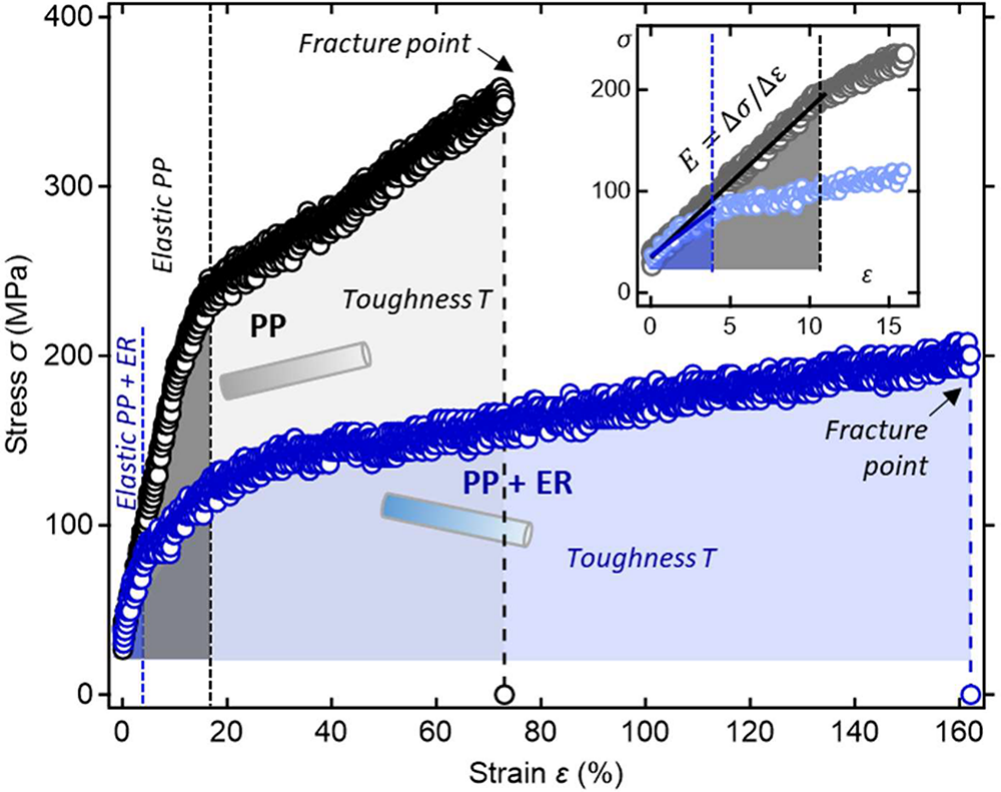
Representative engineering stress vs strain (σ vs ε) curves of single polypropylene (PP) and polypropylene fiber with erucamide (PP + ER); the inset shows an enlarged view of the low strain elastic region with the linear fits from which the Young’s modulus, *E*, was calculated.

Figure 5 shows representative stress–strain curves for PP and PP + ER single fibers and the mechanical parameters averaged over ten fibers per fiber type are listed in Table 2, with the results for the individual fibers listed in Table S8, SI.12. The average diameter was consistent for all the fibers, *d*_f_ = 12.8 μm ± 0.7 μm (PP + ER) and *d*_f_ = 13.0 μm ± 1.1 μm (PP). The stress–strain curves were characteristic for melt-spun polymer fibers.^91^ That is, the stress, σ, increased linearly with strain, ε, reaching an elastic limit at the *yield stress* (σ_y_), followed by a region of nonlinear behavior due to plastic deformation.

Erucamide significantly decreased the elastic limit. As a result, PP + ER appeared more ductile, with the average fracture strain (i.e., elongation at break) ε = 162 ± 6%, appreciably exceeding that of the PP fiber (ε = 71 ± 6%). Furthermore, the erucamide presence resulted in lower *tensile strength* (at the fracture point), i.e., σ = 192 ± 6 MPa for PP + ER, which is smaller than σ = 344 ± 25 MPa for PP. The *yield strength* of PP + ER is σ_y_ = 80 ± 6 MPa at ε = 4%, whereas for PP the nonlinear behavior emerged only above ε = 10 ± 2% with σ_y_ = 201 ± 22 (Figure 5). The average *Young’s modulus* (eq S13) was *E* = 1.5 ± 0.3 GPa for PP and *E* = 1.2 ± 0.2 GPa for PP + ER, suggesting that erucamide enhanced fiber softness often defined as inversely related to the stiffness of the material.^92^

The area enclosed by the σ–ε curve is a measure of the *toughness*, defined as the amount of energy that fiber can absorb before fracture. The fiber average toughness (eq S14), *T* = 188 ± 8 Jm^3–^ (PP + ER) and *T* = 153 ± 16 Jm^3–^ (PP), demonstrates that erucamide promoted fiber ductility.

To account for the change in the fiber cross-section upon increased strain, the *true* stress and strain were calculated according to eq S11 and eq S12, respectively, in SI.03. The difference between the engineering and true stress–strain curves was negligible within the elastic region but pronounced in the plastic region as a result of polymer chain unfolding upon shear (Figure S16), particularly for PP + ER due to its superior ductility (Table 2).

Overall, these single-fiber mechanical parameters corroborated with those reported for melt-spun polypropylene fibers.^91,93^ For instance, the tensile strength of polypropylene fibers is reported to be in the range 50–600 MPa, fracture strain 50–600%, and Young’s Modulus 0.5–3 GPa.^93−95^

### Linking the Single-Fiber Crystal Structure with Elastic Properties: Effects of Erucamide

**Figure 6:**
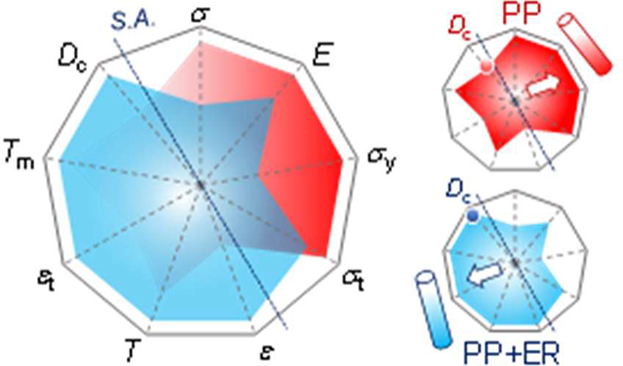
A radar chart of the single-fiber crystal structure-elastic property (CS-EP) profile, giving an overview of the relative differences between PP (red) and PP + ER (blue) in the following parameters: engineering tensile strength (σ), Young’s modulus (*E*), yield strength (σ_y_), true tensile strength (σ_t_), engineering elongation at break (ε), toughness (*T*), true elongation at break (ε_t_), melting temperature (*T*_m_), and the degree of crystallinity (*D*_c_). In plotting the chart, which appears as a nonagon with nine dimensionless axes/spokes all on a scale of 0–1, each of the nine parameters is normalized with respect to the maximum values registered across the two fiber types (PP and PP + ER; cf. Table 2). Then lines are drawn to join the data value on each of the axes. A dashed line across the nanogon, labeled as S.A. (“*softness axis*”), partitions the parameters such that the fiber with higher values of those on the left appears “softer”. The radar charts on the right-hand side show the individual CS-EP profiles for the PP and PP + ER single fiber, respectively, with the crystallinity degree (*D*_c_) axis highlighted.

The high spatial resolution (i.e., 60 nm beam size and 100 nm scan stepsize) of synchrotron nXRD has yielded nanostructural information on a single-fiber level, revealing the bimodal polymer chain orientation (along the fiber axis and cross-section, respectively; cf. Figure 3) and subtle spatial variations in the fiber crystal structure (cf. Figure 4). The overriding feature, however, is that the presence of erucamide in the fiber appreciably affected its crystal structure, resulting in a higher crystalline fraction within the polymer matrix (cf. Figure 1 and 4; also DSC confirmation in Figure S15) and promoting the prevalence of the daughter lamellae along the fiber length (cf. Figure 3). Complementary single-fiber uniaxial tensile tests have shown that erucamide significantly affected fiber mechanical properties, decreasing fiber tensile strength and stiffness but enhancing fiber toughness, fracture strain, and ductility—properties associated with fiber “softness”. The radar chart in Figure 6 gives an overview of the relative differences and correlation between different parameters characterizing the crystal structure (*D*_c_ and *T*_m_) and the mechanical properties (σ, *E*, σ_y_, σ_t_, ε, *T*, and ε_t_) (cf. Table 2). The dashed line across the nanogon, labeled as S.A. (“*softness axis*”), partitions the parameters such that the PP + ER fiber with higher values of those on the left appears “softer”.

Erucamide, widespread in industrial polymer processing and known as a “slip additive”, has been previously understood to play a critical role in modifying polymer fiber surface chemistry and morphology. However, how would erucamide additives facilitate enhanced fiber crystallization as observed? How could we correlate the enhanced single-fiber crystallinity with the enhanced single-fiber toughness and ductility?

In fiber melt spinning, crystallization occurs under tension between the polymer melt extruder and the winder, facilitating preferential stress-induced crystallization and orientation of lamellar crystallites.^67,78−80^ The erucamide fraction embedded within the polymer matrix is situated within the amorphous region around the crystalline lamellae.^22^ We suggest that erucamide could lubricate and thus facilitate interlamellar slip during fiber crystallization, in a mechanism similar to the classic boundary lubrication mediated by surfactants, making it easier for chain disentanglement and thus arrangement and orientation of the crystallites and amorphous macromolecules. This would result in larger crystallites and a higher number of chains aligned along the fiber length within the ordered fraction of the microfibrils, compared to pure PP fibers without erucamide.^81−83^ This interpretation of erucamide facilitating polymer chain disentanglement and resulting in larger polymer crystallites is consistent with larger crystallite sizes and more polymer chains within the crystalline regions for PP + ER (Table S2, SI.04).

In contrast, a previous study reported that the erucamide content had no obvious effect on the degree of crystallinity of isotactic polypropylene films.^22^ A possible explanation for this discrepancy might be related to different processing for polymer *films* and polymer *fibers*. Unlike stress-induced crystallization and orientation of crystallites in fiber melt spinning, in the case of polymer films, the absence of the external stress facilitates spherulitic morphology with radial symmetry.^78,79^ The fact that erucamide had little effect on the size of the daughter lamellae composed of laterally oriented chains, while having a significant effect on the size of the parent lamellae composed of chains oriented along the fiber axis (i.e., along the stress direction during melt spinning), provides evidence for the mechanism underpinning the erucamide action, i.e., it is associated with the stress-induced crystallization. We also note that, whilst plasticizer addition has been reported to improve ductility of polymer films, it reduced the film melting point, in contrast to the effect of erucamide on the PP fibre observed here.^96^

Such a role of interlamellar erucamide in stress-induced fiber crystallization may also offer an explanation to the observations from the single-fiber mechanical tests. In these, deformation of the semicrystalline polymers under the external force occurs via crystal fragmentation and recrystallization processes based on a range of micromechanisms, respectively characteristic for the crystalline phase (e.g., crystallographic slip, mechanical twinning, and stress induced transformations) and for the amorphous phase (e.g., interlamellar shear, lamella separation, and stack rotations). These processes occur simultaneously as a result of the crystalline and amorphous phases being covalently bonded via polymers acting as “*tie molecules*”.^50,64,81,97^

While crystallographic slip (i.e., chain slip) in the (110), (010), and (100) planes is a predominant deformation mode for α-isotactic polypropylene crystals,^98,99^ the amorphous phase—where the main precursors of fracture (e.g., cavities or voids) develop^98,100^—can be expected to play an important role in fiber plastic deformation. Under tension along the fiber axis, the interlamellar (i.e., interfibrillar) *shear* and *separation* are the predominant modes of the amorphous deformation, leading to the elongation of the interlamellar-chains.^77,82,99,101^ As a result, the friction between shearing microfibrils and between the tie molecules is considered the main source of fiber resistance to deformation.^77^ Under tension, structural evolution may occur via daughter lamellae rotation toward the direction of the tensile stress and its partial destruction, followed by fragmentation and reordering of both the parent and daughter lamellae, and finally recrystallization along the tensile direction.^64^

Erucamide has been previously shown to decrease adhesion and friction of semicrystalline polymers and fibers.^1,11−14^ The mechanism is underpinned by its structural organization in the lamellae composed of bilayers on the polymer/fiber surface, with the hydrophobic tails pointing outward, facilitating inter-erucamide layer sliding. We suggest that the “slip additive” nature of erucamide could also account for its effects on the single-fiber mechanical properties. That is, erucamide situated at the interfaces surrounding the polypropylene crystallites^22^ can reduce the stress from microfibril shear displacement and reduce the stress from the deformation of the interlamellar macromolecular network via reduction of friction between the neighboring microfibrils, facilitating their sliding motion past each other.

Furthermore, it is likely that erucamide can facilitate rotation and structural rearrangement of daughter and parent lamellae under tensile stress. Concurrently, since erucamide occupies a fraction of inter-lamellar region, it is plausible that the number of interfibrillar tie molecules is lower in PP + ER than in PP. In contrast, in the PP fiber, the stronger inter-fibrillar chain entanglement contributes to higher internal friction and resistance to deformation, resulting in greater tensile strength and faster sample failure (i.e., smaller elongation at break).^77,83^

It has been previously reported that erucamide could promote the β-polypropylene crystalline form^102,103^ and that polymer mixtures with increased β-polypropylene content exhibited lower tensile strength, higher elongation and toughness when compared to α-polypropylene. This is consistent with our results on the single fibers. Furthermore, we note that Young’s modulus was previously reported to be independent of the crystallite fraction or the β-polypropylene prevalence. This is consistent with our proposed mechanism above, that is, the difference in the single-fiber stiffness could be attributed to erucamide acting as a slip additive between the polymer chains in the PP + ER fiber which would facilitate sliding between crystallites upon fiber stretching.^104^

## Conclusions

Previous reports concerning erucamide-loaded polymer materials have mainly focused on the polymer surface properties (i.e., friction, surface roughness, morphology, and adhesion).^8−13^ Several studies examined the mechanism behind erucamide solubility and diffusion through the polymer film matrix, as a function of the additive concentration and polymer chemistry.^19,22^ The understanding of the effect of erucamide on the polymer crystal structure and mechanical properties remained unexplored, specifically with regards to polymer fibers, which are highly relevant to personal care products. Here, we report a single-fiber synchrotron nanofocused X-ray Diffraction (nXRD) study on the effects of erucamide on the fiber crystal structure along the fiber length and across the fiber width.

In our experimental approach, advanced quantitative physicochemical methods have been combined to obtain single-fiber nXRD and mechanical test results, enabling a direct structure–property correlation. This has facilitated physical insights: we propose that erucamide promoted crystallization via mediating slip at the interfaces between crystallites and amorphous domains during stress-induced single-fiber crystallization. This would serve to encourage polymer chain disentanglement and mobility, thus mediating rotation, reordering, and recrystallization of crystal lamellae as well as the sliding motion of neighboring microfibrils. While encouraging crystallinity as we observed, the erucamide presence also affected fiber structural evolution upon uniaxial tensile stress, decreasing the fiber stiffness and promoting the toughness and ductility. The role of interlamellar erucamide in stress-induced fiber crystallization may also offer an explanation to the observations from the single-fiber mechanical tests. That is, erucamide situated at the interfaces surrounding the polypropylene crystallites can reduce the stress from microfibril shear displacement and reduce the stress from the deformation of the interlamellar macromolecular network via reduction of friction between the neighboring microfibrils, facilitating their sliding motion past each other. Our results from this study and our previous studies^1,14^ have demonstrated the role of erucamide in optimizing polypropylene crystallinity, mechanical properties, and surface properties on a single fiber level. It serves to explain the molecular origin of how such an additive could enhance fiber feel and handling, both highly relevant to manufacturing and widespread application of fibrous materials.

To gain more insights into the specific mechanisms behind crystallization and plastic deformation of erucamide-loaded polypropylene fibers, future nXRD studies could probe the *in situ* formation and evolution of fiber crystal structures during melt spinning and upon further uniaxial stretching (i.e., drawing).

## Experimental Methods

Details are described in the Supporting Information Section: SI.01 Materials and Methods; SI.03 Measurement of the tensile strength and Young’s modulus of a single fiber; SI.11 Differential Scanning Calorimetry.

## Supporting Information Available

SI.01 Materials and Methods; SI.02. XRD peak analysis; Fiber unit cell parameters; Degree of crystallinity; SI.03 Measurement of the tensile strength and Young’s modulus of a single fiber; SI.04 nXRD results on sing-fiber crystal structure; SI.05 Crystal structure; SI.06 Crystal orientation with respect to fiber axis; SI.07 The off-equatorial and off-meridional reflections; SI.08 Scherrer analysis; SI.09 Crystallite size and degree of crystallinity along fiber length; SI.10 Crystallite size and degree of crystallinity across fiber width; SI.11 Differential Scanning Calorimetry; SI.12 Mechanical properties (PDF)
