# Supplementary material for: Effects of Erucamide on Fiber “Softness”: Linking Single-Fiber Crystal Structure and Mechanical Properties
Source: ACS Nano. 2024 Feb 9;18(7):5940–50. doi: 10.1021/acsnano.4c00114 (PMC10883039; doi:10.1021/acsnano.4c00114)
Supplement: Supplementary file 1 — nn4c00114_si_001.pdf [file nn4c00114_si_001.pdf]

Supplementary Information

Effects of erucamide on fibre "softness": *Linking single-fibre crystal structure and mechanical properties*

Dajana Gubała [1], Anna Slastanova [1,a], Lauren Matthews [1,2,b], Luisa Islas [1,c], Patryk Wąsik [1,2,d], Fernando Cacho-Nerin [3,e], Dario Ferreira Sanchez [4], Eric Robles [5], Meng Chen [6], and Wuge H. Briscoe [1]*

[1] School of Chemistry, University of Bristol, Cantock's Close, Bristol BS8 1TS, UK

[2] Bristol Centre for Functional Nanomaterials, HH Wills Physics Laboratory, University of Bristol, Bristol BS8 1TL, UK

[3] Diamond Light Source, Diamond House, Harwell Science and innovation Campus, Didcot, Oxfordshire, OX11 0DE, UK

[4]Paul Scherrer Institut, Forschungsstrasse 111, Villigen PSI, 5232, Switzerland

[5] Household Care Analytical, Procter & Gamble Newcastle Innovation Centre, Whitley Road, Longbenton, Newcastle, NE12 9TS, UK

[6] Procter & Gamble Technology (Beijing) Co., Ltd., 35 Yu'an Rd, Shunyi District, Beijing 101312, China

Present address:

[a] Purdy & Figg Ltd, Eastman Way, Hemel Hempstead, HP2 7FW, England

[b] ESRF, The European Synchrotron, 71 avenue des Martyrs, Grenoble 38043, France

[c] Johnson Matthey Technology Centre, Blounts Ct Rd, Sonning Common, Reading, RG4 9NH

[d] National Synchrotron Light Source II, Brookhaven National Laboratory, Upton, New York 11973, USA

[e] AVS, Pol. Ind. Sigma, Xixilion kalea 2, Bajo, Pabellón 10, 20870 Elgoibar, Gipuzkoa, Spain

** E-mail: wuge.briscoe@bristol.ac.uk; Tel: +44 (0)117 3318256*

SI.01 Materials

Polymer fibres were donated by Procter & Gamble (Beijing). Samples were single fibres of polypropylene (PP), a homopolymer designed by ExxonMobil™ specifically for spunbonded nonwovens, with one sample also containing 1.5 wt% of erucamide ($C_{22}H_{43}NO$; Figure 1). The erucamide was introduced prior to fibre extrusion by mixing the pure polypropylene resin with masterbatch (composed of 10 wt% erucamide and 90 wt% polypropylene) in an 85:15 ratio, yielding 1.5 wt% erucamide per fibre. The fibres also contained 1 wt% titanium dioxide (TiO_2), added *via* premixing with the PP resin before fibre spinning. The fibres, denoted as **PP** and **PP + ER** for the erucamide-loaded fibre, thus differed only in the 1.5 wt% erucamide additive in the PP + ER fibres, with the same PP matrix and fibre extrusion process. For sample preparation, single fibres were carefully removed from fibre bundles with tweezers, avoiding or minimizing any fibre stretching and breakage. Erucamide (Sigma Aldrich, >85%) was re-crystallised once from acetone (Sigma Aldrich, 99.5%) prior to being transferred to glass capillaries for XRD measurements.

Methods

nXRD experimental setup; XRF mapping for fibre alignment; μ–XRD measurements for control samples

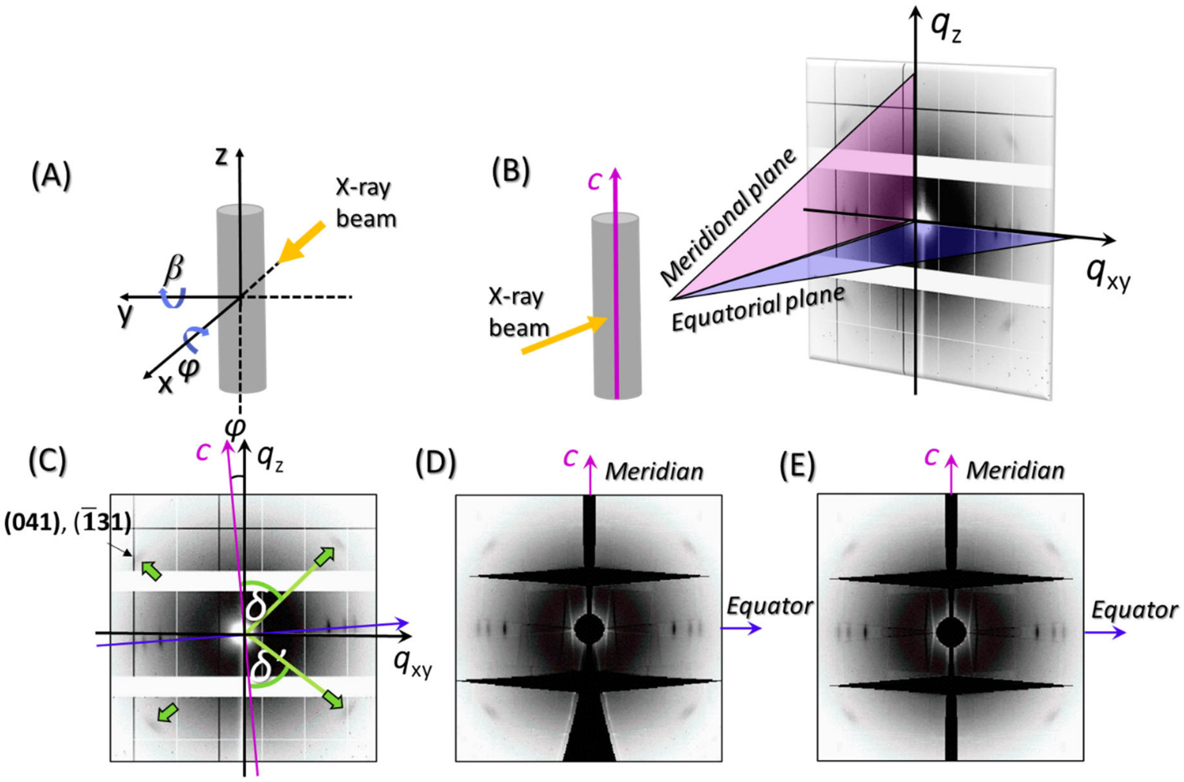

Figure S1 (A) Schematic representation of rotational angles of a single fibre with respect to the direction of the incident X-ray: β (xz plane) and φ (yz plane). (B) Schematic representation of the geometry of X-ray diffraction by a vertically aligned fibre (fibre *c*-axis shown in pink), showing the meridional (or vertical) plane (q_z) and the equatorial (or horizontal) plane (q_{xy}) scattering vectors on the 2D detector plane. (C) 2D XRD pattern obtained for a fibre titled by the angles β and φ with respect to the incident beam, and the fibre c-axis can be found at the centre of the off-equatorial and off-meridional Bragg reflections (4.07 Å, [(041)/($\bar{1}$31)]) (indicated by the green arrows). The mirror symmetry about the *c*-axis allowed determination of fibre tilt φ. Fibre alignment and Fraser correction [1] were attained *via* measurement of the azimuthal angle φ, defined as the angle between the normal direction (scattering vector q_z) and the fibre axis (pink arrow). β is calculated as $\tan\beta = \frac{\sqrt{4-\lambda^2 s_r^2}}{2\lambda s_r}(cos\,\delta' - cos\,\delta)$, where λ is the X-ray wavelength, s_r is the radius of Polanyi sphere of the reference 041/$\bar{1}$31 reflection,

$s_r = \frac{1}{d_{041/\bar{1}31}}$, and δ and δ' the direction angles measured against fibre meridional axis. Here, φ = 4.5° and β = 7.4°. (D) Corrected fibre diffraction pattern mapped into reciprocal space. (E) A four-quadrant fibre pattern mapped into reciprocal space with the missing information for the lower half copied from the upper half.

The nano-focused XRD experiment was performed at beamline I14 at Diamond Light Source (DLS), Didcot, UK, using an X-ray beam with an energy of 20 keV (λ = 0.619 Å), with an integration time of 30 seconds per frame. The beam size was 60 nm × 60 nm with an angular resolution of 10 millidegrees (dq = 0.02 nm^{-1}) [2]. The experimental setup is schematically shown in Figure S2A, along with the measurement details, in Section SI.01. A single fibre was scanned at four sections (S_1-S_4) along 300 µm of the length, with 3 scans in each section 1 µm apart, with a 100 nm step size in S_1 and a 500 nm step size in S_2-S_4. The crystal structure along fibre width was investigated at three areas (A_1-A_3) marked in orange in S_1. A single fibre was fixed (vertically w.r.t. the incident X-ray beam) on the copper frame of a sample holder using Kapton (poly (4,4'-oxydiphenylene-pyromellitimide)) tape. All the measurements were performed under ambient conditions. The XRD scans were performed in the transmission mode and the intensity was detected with a 285 mm sample-to-detector distance using a 3-module Excalibur detector [3]. Calibration of the detector was performed using the cerium oxide (CeO_2) powder standard.

Optical microscopy followed by X-ray fluorescence (XRF) spatial mapping of TiO_2 particles distributed within the fibre matrix were applied to locate and align the fibre in the nano-focused beam. The fluorescence scan was attained at a scan size ~25-30 µm, exceeding that of fibre diameter (d_f <15 µm), at a step size of 500 nm and an exposure time of 1 s. An example XRF scan is shown in Figure S2C, showing a uniform distribution of TiO_2 particles across the

PP fibre. The apparent fibre diameter values obtained based on the XRF scans were consistent with those attained later *via* FDAS770 Laser Scanning Micrometre (Dia-stron).

For the nXRD measurement to probe the fibre crystal structure along its length and width, the vertically aligned fibre was scanned with the nano-focused beam, 3 times along the lines 1 µm apart (Figure S2B) at each of the four sections (S_1-S_4) along 300 µm of the fibre length. The scans in the first section (S_1) were attained with a 100 nm step size and in S_2-S_4 with a 500 nm step size. Based on the initial scans, the fibre crystal structure was further analysed at three discrete positions in S_1, denoted as A_1-A_3, each of 3 µm in length across the fibre width (Figure S2B).

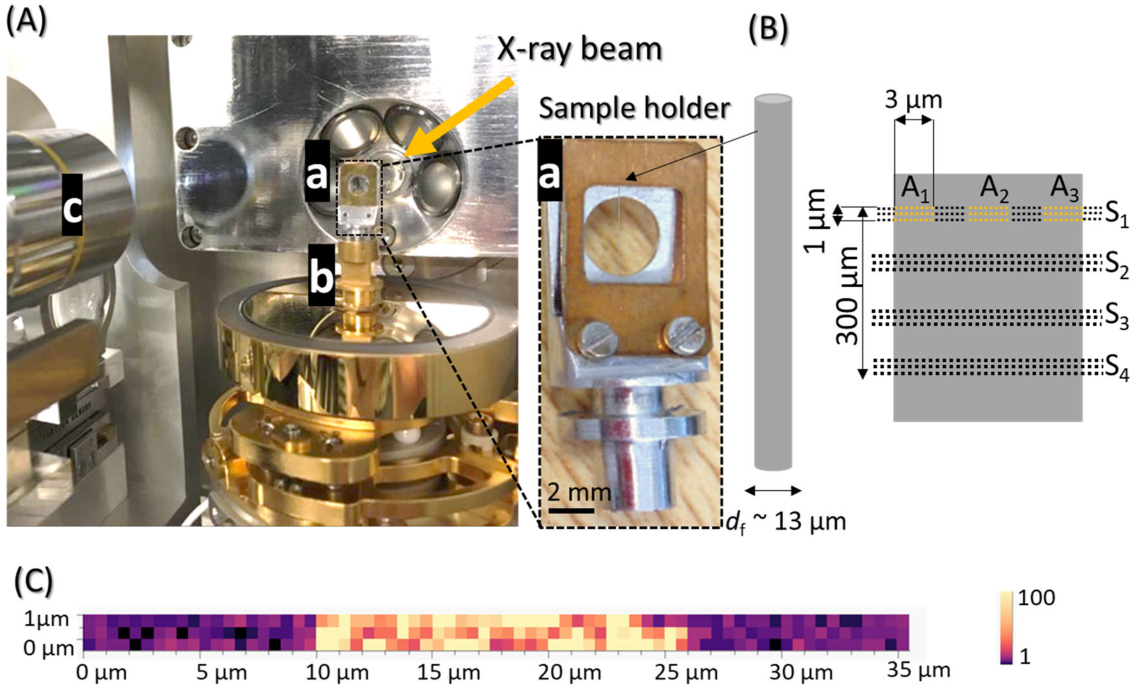

Figure S2 (A) Nano-focused XRD (nXRD) experimental setup at Diamond Light Source beamline I14: **a** - sample holder with vertically aligned fibre fixed on the copper frame; **b** - sample stage; and **c** - detector/camera; (B) schematic representation showing four sections (S_1-S_4) scanned along 300 µm of fibre length, with 3 scans in each section 1 µm apart, with a

100 nm step size in S_1 and a 500 nm step size in S_2-S_4. The crystal structure along fibre width was investigated at three areas (A_1-A_3) marked in orange in S_1; and (C) an example of XRF results obtained for titanium dioxide facilitating single fibre alignment (with a scan size of 35 µm x 1 µm).

The XRD scan for the control erucamide powder sample was attained at microXAS beamline (X05LA), Swiss Light Source (SLS), Paul Scherrer Institute, Villigen, Switzerland, using an X-ray beam with an energy of 17.2 keV (λ = 0.721 Å), with an integration time of 4 s. The beam size was 1 µm × 1 µm and the angular resolution 10 millidegrees (dq = 0.02 nm^{-1}). The diffraction patterns were recorded in the transmission mode using an 8-module Eiger detector (Dectris, Switzerland). Lanthanum hexaboride (LaB_6) powder standard was used to calibrate the detector geometry.

Raw XRD 2D images from DLS and SLS were processed using Dawn [4] and Datasqueeze 3.0 software [5], respectively, involving appropriate masking to remove the detector grid, intermodular gaps (123 pixels, vertical) and overexposed or dead pixels, as well as applying corrections for the diffraction intensity (*i.e.* polarization) and geometric effects associated with the fibre alignment (Figure S1) [1, 6, 7]. The azimuthal integration over 1793 x 2069 pixels generated 1D diffraction curves plotted as intensity (arb. u.) *vs* momentum transfer q (nm^{-1}), as well as azimuthal profiles plotted as intensity (arb. u.) *vs* azimuthal angle φ (°). A baseline corrections was applied in Python using the asymmetric least squares smoothing method [8].

SI.02 XRD peak analysis; Fibre unit cell parameters; Degree of crystallinity

XRD peak analysis was performed in IGOR Pro (WaveMetrics, Portland, USA) using the Multi-peak Fit operation. The XRD profiles were considered composed of Gaussian or Lorentzian

profiles and were fitted to pseudo-Voigt and Gaussian functions. The characteristic lattice spacing (*d*-spacing) could be ascertained according to , **Equation S1**:

$$d = \frac{2\pi}{q}, \quad \textbf{Equation S1}$$

where *q* is a momentum transfer vector defined as:

$$q = \frac{4\pi \sin\frac{2\theta}{2}}{\lambda} \quad \textbf{Equation S2}$$

and the crystallite size L_a, in the direction of *hkl* plane (*i.e.* perpendicular to the *hkl* plane), could be obtained using Scherrer's equation [9-17]:

$$L_a = \frac{2\pi K}{\Delta Q}, \quad \textbf{Equation S3}$$

where *K* is the Scherrer constant (*i.e.* a shape factor) of order unity depending on the actual shape of the crystalline (here *K* = 1). The number of vertically aligned polymer chains ordered in the direction of the planes responsible for the formation of *hkl* Bragg reflection was estimated as:

$$m = \frac{L_a}{d}. \quad \textbf{Equation S4}$$

The monoclinic unit cell parameters (*a, b, c, β, α = γ = 90°*) were calculated based on the fitted interplanar spacing (*d*-spacing) values corresponding to the crystal planes of Miller indices (*hkl*) according to Equation **S5** [18].

$$\frac{1}{d^2} = \frac{h^2}{a^2 sin^2\beta} + \frac{k^2}{b^2} + \frac{l^2}{c^2 sin^2\beta} - \frac{2hl\cos\beta}{ac sin^2\beta} \quad \textbf{Equation S5}$$

The unit cell volume (*V*) was calculated according to Equation **S6** and crystalline density (*ρ*) , Equation **S7**:

$$V = abc \sin\beta \qquad \textbf{Equation S6}$$

$$\rho = \frac{ZM_w}{VN_A}, \qquad \textbf{Equation S7}$$

where Z is the total number of monomers per unit cell (for polypropylene Z = 12), M_w the molecular weight, and N_A the Avogadro number [18, 19].

The degree of crystallinity was calculated as:

$$D_c = \frac{A_c}{A_c + A_a}, \qquad \textbf{Equation S8}$$

where A_c and A_a are integrated intensities under fitted crystalline part (*i.e.* Bragg peaks) and amorphous halo, respectively (*cf*. Figure 2 in main text).

SI.03 Measurement of the tensile strength and Young's modulus of a single fibre

The tensile strength measurements were conducted using an LEX820 Linear Extensometer with a Laser Diffraction System (Dia-stron). To ensure that the fibre axis was aligned coaxially with the line of action of the testing machine, the single fibre was mounted within the grooves on a pair of plastic tabs. Single fibres were secured with a drop of glue (Loctite Super Glue for all plastics, Rapid Electronics Ltd) placed on each side of the sample holder and dried overnight. Ten fibres each for PP and PP + ER were measured. Prior to each measurement, the fibre diameter was attained with FDAS770 Laser Scanning Micrometre (Dia-stron) (Figure S3A). The tensile strength measurements were run at the pulling speed of 0.1 mm s^{-1}, with a gauge force of 0.005 N and a gauge length of 12 mm (Figure S3B) corrected *via* pre-tensioning of the fibres [20]. The *engineering tensile strength, σ*, calculated as:

$$\sigma = \frac{F}{\pi\left(\frac{d}{2}\right)^2}, \qquad \textbf{Equation S9}$$

where *F* is the load at the failure and *d* is the fibre diameter. σ was plotted as a functionn of the *engineering strain,* ε*:*

$$\varepsilon = \frac{L-L_0}{L_0}, \quad \textbf{Equation S10}$$

where L_0 and *L* are the original and final lengths of the fibre, respectively.

To account for the changes in the fibre cross-section and length upon elongation, the *true* stress σ_t and strain ε_t were calculated according to Equation **S11** and Equation S12, respectively:

$$\sigma_t = \sigma(\varepsilon + 1), \quad \textbf{Equation S11}$$

$$\varepsilon_t = \ln(\varepsilon + 1) \quad \textbf{Equation S12}$$

The Young's modulus *E* was determined from the linear region of the engineering tensile stress *vs* engineering tensile strain curve according to Equation **S13**,

$$E = \frac{\Delta\sigma}{\Delta\,\varepsilon} \quad \textbf{Equation S13}$$

and toughness, *T,* was determined as the area under the stress-strain curve according to Equation S14:

$$T = \int_0^{\varepsilon} \sigma d\varepsilon. \quad \textbf{Equation S14}$$

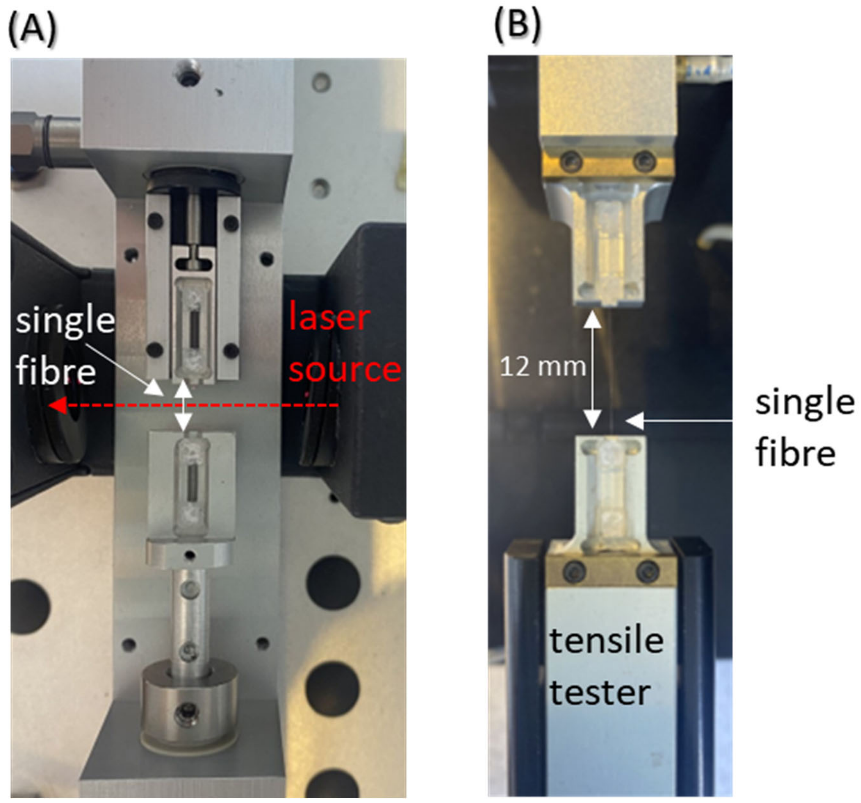

Figure S3 Experimental set-up for (A) the fibre diameter measurement (the red dashed arrow indicates the laser beam direction) prior to (B) the tensile strength measurement.

SI.04 nXRD results on sing-fibre crystal structure

Table S1 Fitted crystal structural parameters for polypropylene (PP) and polypropylene fibre with erucamide (PP + ER): average peak position *q*, crystal planes (*hkl*), and *d*-spacing (Å). Two specimens per fibre type were studied. The errors for the *d*-spacing were calculated *via* the error propagation method based on the uncertainty of the peak position, also considering the angular resolution of the instrument, which is smaller than 0.1 Å. The errors calculated as the

standard deviation between the 4 sections of two fibres per sample batch yielded less than 0.06 nm^{-1} for the peak position *q*.

Fibre	*Average q (nm^{-1})*	*hkl*	*d*-spacing (Å)								
			Fibre 1				***Fibre 2***				***Average d*-spacing (Å)**
			S_1	S_2	S_3	S_4	S_1	S_2	S_3	S_4	
**PP**	9.98	110	6.30	6.29	6.30	6.29	6.31	6.30	6.30	6.30	6.30 ± 0.01
	11.91	040	5.30	5.27	5.27	5.27	5.30	5.27	5.27	5.27	5.28 ± 0.01
	13.08	130	4.82	4.80	4.80	4.80	4.82	4.80	4.79	4.79	4.80 ± 0.01
	14.92	111	4.22	4.20	4.23	4.20	4.20	4.21	4.22	4.21	4.21 ± 0.01
	15.42	$041/\bar{1}31$	4.08	4.07	4.08	4.07	4.08	4.07	4.08	4.07	4.08 ± 0.01
	17.78	060	3.54	3.54	3.56	3.54	3.53	3.53	3.53	3.53	3.53 ± 0.01
**PP + ER**	9.97	110	6.31	6.30	6.30	6.30	6.31	6.3	6.3	6.3	6.30 ± 0.01
	11.93	040	5.30	5.26	5.26	5.26	5.29	5.26	5.26	5.26	5.27 ± 0.02
	13.10	130	4.82	4.79	4.80	4.79	4.81	4.79	4.79	4.79	4.80 ± 0.01
	14.94	111	4.21	4.19	4.21	4.20	4.21	4.2	4.21	4.21	4.21 ± 0.01
	15.45	$041/\bar{1}31$	4.08	4.06	4.07	4.06	4.08	4.07	4.07	4.06	4.07 ± 0.01
	17.75	060	3.54	3.54	3.54	3.54	3.54	3.56	3.56	3.54	3.54 ± 0.01

Table S2 Fitted structural parameters for polypropylene fibre (PP) and polypropylene fibre with erucamide (PP + ER) at the four sections (S_1-S_4; *cf.* Figure S2) along the fibre length: the (minimum) crystallite domain size (L_a) in the direction perpendicular to the *(hkl)* plane, and the number of vertically aligned polymer chains (*m*) oriented parallel to the plane (*hkl*) for the reflection. The fitted structural parameters corresponding to the equatorial (parent lamellae) and meridional (daughter lamellae) contributions to (110) reflection are denoted as 110 *eq.* and 110 *m.*, respectively. The errors for L_a are calculated *via* the error propagation using the uncertainties in *FWHM* (Δq). The errors for *m* calculated as a combination of the uncertainties in *d* and L_a are all smaller than 1.

**Sample**		***hkl***	**L_a (Å)**				***m***			
			S_1	S_2	S_3	S_4	S_1	S_2	S_3	S_4
**PP**	***Fibre 1***	110 *eq.*	173 ± 7	170 ± 15	177 ± 39	176 ± 14	27	27	28	28
		110 *m.*	108 ± 1	94 ± 1	128 ± 2	94 ± 1	17	15	20	15
		040	152 ± 24	174 ± 30	165 ± 27	169 ± 27	29	33	31	32
		130	107 ± 24	105 ± 26	103 ± 25	115 ± 28	22	22	22	24

		111	111 ± 54	100 ± 53	120 ± 46	102 ± 47	26	24	28	24
		041/$\bar{1}31$	106 ± 34	105 ± 35	96 ± 22	110 ± 33	26	26	23	27
		060	50 ± 28	53 ± 31	49 ± 32	53 ± 31	14	15	14	15
	Fibre 2	110 *eq.*	178 ± 10	180 ± 10	174 ± 9	172 ± 10	28	29	28	27
		110 *m.*	97 ± 1	102 ± 1	102 ± 1	86 ± 1	15	16	16	14
		040	173 ± 27	168 ± 31	171 ± 27	166 ± 27	33	32	32	31
		130	111 ± 27	108 ± 21	120 ± 26	98 ± 19	23	22	25	20
		111	99 ± 60	104 ± 34	121 ± 26	107 ± 49	23	25	29	25
		041/$\bar{1}31$	115 ± 44	104 ± 22	100 ± 20	106 ± 31	28	26	25	26
		060	41 ± 4	34 ± 2	34 ± 2	34 ± 2	12	10	10	10
**PP + ER**	***Fibre 1***	110 *eq.*	242 ± 7	248 ± 9	240 ± 8	235 ± 8	39	39	38	37
		110 *m.*	97± 1	101 ± 1	108 ± 1	107 ± 1	15	16	17	17
		040	189 ± 27	194 ± 30	194 ± 30	188 ± 30	36	37	37	36
		130	122 ± 25	153 ± 32	132 ± 30	138 ± 32	25	32	28	29
		111	142 ± 52	118 ± 39	138 ± 50	130 ± 50	34	28	33	31
		041/$\bar{1}31$	134 ± 33	164 ± 36	139 ± 31	152 ± 36	33	40	34	37
		060	90 ± 25	90 ± 25	84 ± 23	102 ± 33	26	26	24	29
	Fibre 2	110 *eq.*	255 ± 1	230 ± 12	249 ± 9	243 ± 10	40	36	40	39
		110 *m.*	105 ± 1	97 ± 1	112 ± 1	107 ± 1	17	15	18	17
		040	210 ± 30	252 ± 35	239 ± 32	215 ± 19	40	48	45	48
		130	149 ± 47	192 ± 41	157 ± 33	144 ± 27	31	40	33	40
		111	158 ± 25	158 ± 14	149 ± 39	132 ± 19	37	38	35	38
		041/$\bar{1}31$	141 ± 14	148 ± 8	140 ± 21	146 ± 14	35	36	34	36
		060	115 ± 68	61 ± 10	61 ± 10	39 ± 20	33	17	17	11

SI.05 Crystal structure

SI.05 Crystal structure – fibre’s replicates

Figure S5 shows the representative diffractograms obtained for the the replicates of polypropylene fibre (PP, Fibre 2) and polypropylene fibre with erucamide (PP + ER, Fibre 2). The 2D diffraction patern for PP + ER closely resembled that of PP and for both fibres all Bragg reflections were assigned solely to isotactic α-polypropylene. Furthermore, the position of reflections corresponding to polypropylene was unaffected by the presence of the erucamide, which is in agreement with our previous findings for bundles of fibres [21].

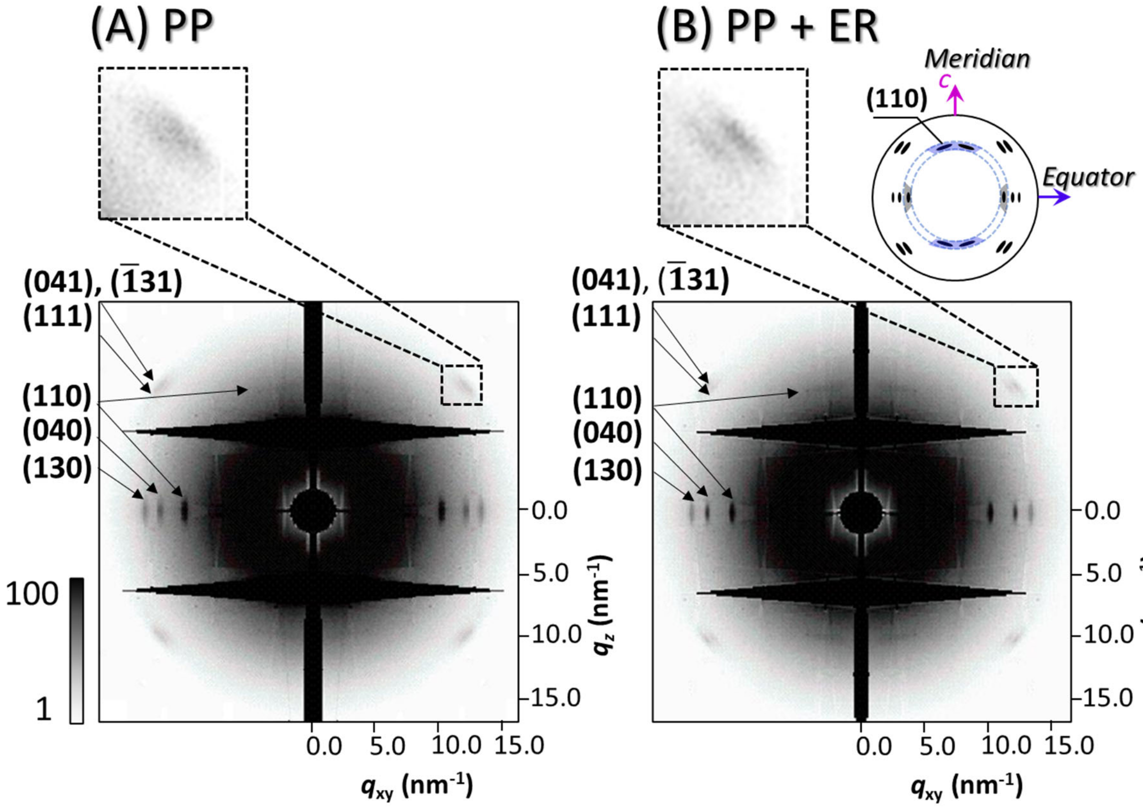

Figure S4 Fibre 1 XRD diffractograms of (A) a polypropylene single fibre (PP) and (B) a polypropylene single fibre with erucamide (PP + ER). The darker colour indicates higher intensity. The lines point to the Bragg reflections assigned to isotactic α-polypropylene. The enlarged images at the top show the off-meridional and off-equatorial Bragg reflections. The schematic representation of a 2D XRD fibre pattern, showing (110) Bragg reflections on the fibre equator (grey) and near the meridian (blue) (110) for clarity.

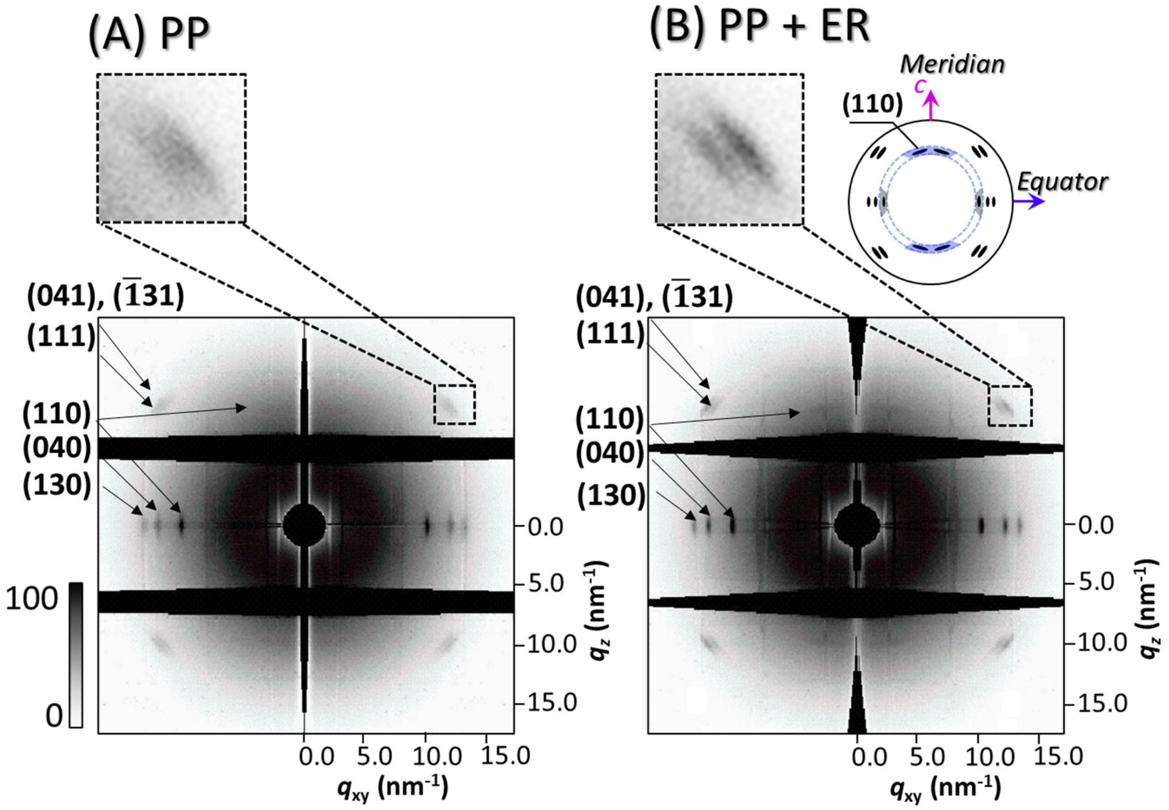

Figure S5 XRD patterns of fibre's replicates (A) pure polypropylene fibre (PP, Fibre 2), (B) polypropylene fibre with erucamide (PP + ER, Fibre 2). The darker colour indicates higher intensity. The lines point to reflections assigned to isotactic α-polypropylene. The enlarged images show off-meridional and off-equatorial reflections obtained for PP *vs* PP + ER.

SI.06 Crystal orientation with respect to fibre axis

Figure S6 shows the azimuthal intensity distribution of the equatorial and near-meridional contributions to (110) reflection (q band of 9-11 nm^{-1}) obtained for fibre replicated PP (Fibre 2) and PP + ER (Fibre 2).

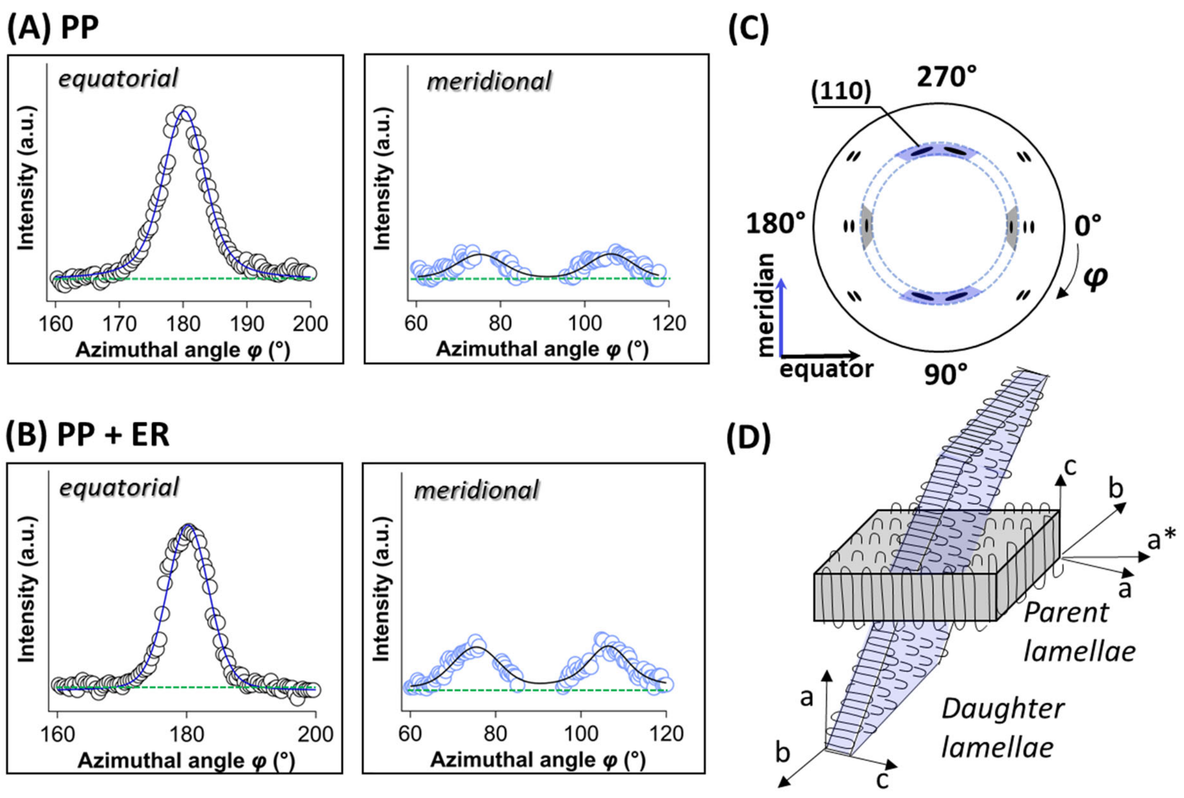

Figure S6 Representative azimuthal intensity distributions of equatorial (φ = 180° ± 30°) and near-meridional (φ = 270°± 30°) (110) reflections (in the q range of 9-11 nm^{-1}) obtained for: (A) polypropylene fibre (PP, Fibre 2) and (B) polypropylene fibre with erucamide (PP + ER, Fibre 2); (C) Schematic representation of the 2D XRD pattern, with the azimuthal region corresponding to equatorial reflections in grey and the azimuthal region corresponding to near-meridional reflections in blue; (D) Schematic representation of the structural model of the bimodal orientation composed of the daughter lamellae, corresponding to near-meridional reflections on the 2D pattern, growing from the parent lamellae, corresponding to equatorial reflections on the 2D pattern. The a, b, and c represent the PP monoclinic unit cell axes and a^* is the component of a axis oriented perpendicular to c and b axes, and represents growth direction of parent lamellae. The a-axis and c-axis of parent lamellae correspond to c-axis and a-axis of the daughter lamellae, respectively.

SI.07 The off-equatorial and off-meridional reflections

The micro-focused X-ray Diffraction data shown in Figure S7 were attained at microXAS beamline (X05LA), Swiss Light Source (SLS), Paul Scherrer Institute, Villigen, Switzerland. The fibre samples were irridatied with micro-focused beam of 1 x 1 µm in size, with the beam energy of E = 17.2 keV (λ = 0.721 Å). Vertically aligned single PP and PP + ER fibres were investigated along 300 µm in fibre length, while the sample was rotated around its axis. The application of 8-module detector, with the intermodular gaps along fibre meridian and equator excluded the important contributions corresponding to the (110), (040) and (130) crystal planes compromising the overall crystal structure profiles. Nevertheless, since the off-equatorial and off-meridional reflections consistent with periodic distance yielding at d = 4.21 Å and d = 4.08 Å (d = 4.07 Å for PP + ER), that corresponds to (111) and (041/131) crystal planes respectively, were unsusceptible to the alignment of detector modules, we were able to confirm that the appearance of the Bragg peaks was different for PP and PP + ER samples as a result of stronger scattering from the crystalline fraction of semicrystalline polymer from the fibre with erucamide masterbatch, suggesting erucamide facilitating a higher crystalline order.

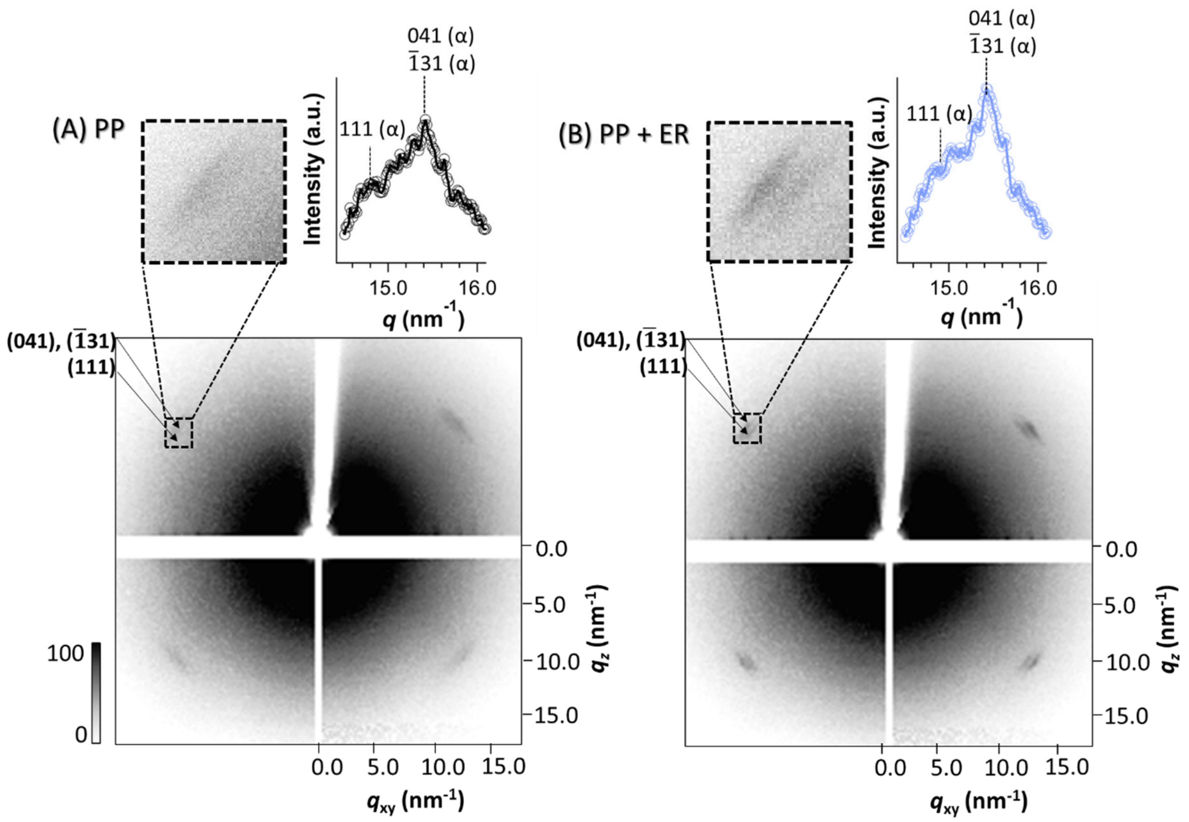

Figure S7 Example XRD patterns of (A) polypropylene fibre (PP) and (B) polypropylene fibre with erucamide (PP + ER), obtained at microXAS beamline (X05LA), Swiss Light Source (SLS), Paul Scherrer Institute, Villigen, Switzerland. The enlarged views show off-meridional and off-equatorial reflections.

SI.08 Scherrer analysis

The crystallite domain size, L_a, and the number of vertically aligned polymer chains, *m*, for PP and PP + ER obtained from different Bragg peaks are shown in Figure S8. The fibres with erucamide exhibited a larger crystallite domain size. The separation of the meridional and equatorial contributions to the (110) Bragg reflection allowed the assessment of the effect of erucamide on the structural order within the crystallites of bimodal orientation. The results obtained from the Scherrer analysis show that erucamide promoted the crystal growth in the direction corresponding to meridian, with an average parent lamellae crystal size of 243 ± 8

Å for PP + ER and 175 ± 3 Å for PP, respectively (Figure S8, Table S3). The additive had a negligible effect on the size of the daughter lamellae, with a crystallite size of 104 ± 5 Å for PP + ER and 101 ± 12 Å for PP, respectively (Figure S8, Table S3). However, it promoted the growth of the daughter lamellae, with the average relative content of the daughter to the parent lamellae across four studied sections (S_1-S_4) along the fibre length valuing at 16 ± 1% (daughter) to 84 ± 1% (parent) for PP + ER and 11 ± 1% (daughter) to 89 ± 1% (parent) for PP (Figure S8, Table S3).

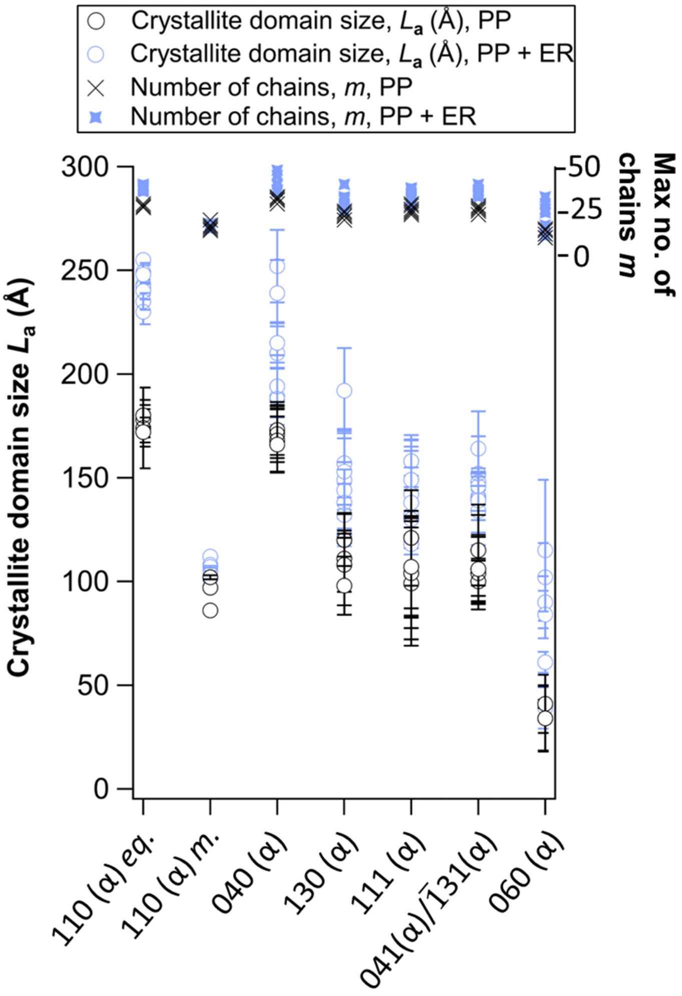

Figure S8 The crystallite domain size (L_a) and the number of vertically aligned polymer chains (m) ordered in direction of the plane (hkl) estimated from the analyses using different Bragg

reflections for the pure PP fibre (PP) and the PP fibre with 1.5 wt% erucamide (PP + ER) measured at the four sections (S_1-S_4) across fibre length, for two fibres per sample.

Table S3 Relative content of the parent and daughter lamellae based on the relative peak intensity of the equatorial and meridional contributions to the (110) Bragg reflection, for the PP fibre and the PP fibre with 1.5 % erucamide (PP + ER) at four sections (S_1-S_4) along the fibre length.

Sample	Lamellae	Relative content (%)							
		Fibre 1				***Fibre 2***			
		**S_1**	**S_2**	**S_3**	**S_4**	**S_1**	**S_2**	**S_3**	**S_4**
**PP**	***parent***	90	87	89	90	90	88	88	90
	daughter	10	13	11	10	10	12	12	10
**PP + ER**	***parent***	85	84	84	84	85	83	83	82
	daughter	15	16	16	16	15	17	17	18

SI.09 Crystallite size and degree of crystallinity along fibre length

The degree of crystallinity, D_c (Equation S8), in the single fibre was calculated based on the Bragg reflections at the q range of 9.9 – 17.8 nm^{-1} (*cf.* Figure 4, main text). Table S4 lists the degree of crystallinity (D_c) calculated for polypropylene (PP) and polypropylene with erucamide (PP + ER) for two samples per fibre type, across four sections (S1-S4) along the fibre length (*cf.* Figure 4B in the main text).

Table S4 Degree of crystallinity (D_c) calculated for polypropylene (PP) and polypropylene with erucamide (PP + ER) for two samples per fibre type, across four sections (S_1-S_4) along the fibre length. The average values were calculated as the weighted arithmetic mean, to account for the larger number of points scanned for section S_1 (step size of 100 nm) than for sections S_2-S_4 (step size of 500 nm). The errors for D_c were calculated *via* the error propagation method

based on the uncertainties in the integrated intensities under the fitted Bragg peaks (A_c) and the amorphous halo (A_a). The errors for the average D_c were calculated as weighted standard deviations from all the sections studied.

Section:	Degree of crystallinity D_c (%)			
	PP		PP + ER	
	Fibre 1	Fibre 2	Fibre 1	Fibre 2
S_1	31 ± 1	32 ± 1	44 ± 1	49 ± 1
S_2	31 ± 2	32 ± 2	44 ± 3	50 ± 2
S_3	27 ± 2	30 ± 2	45 ± 3	51 ± 3
S_4	25 ± 2	28 ± 1	38 ± 3	51 ± 2
**Average**	30 ± 2	31 ± 1	43 ± 2	50 ± 2

Figure S9 shows the experimental XRD curves obtained for Fibre 1 of PP and PP + ER fibre at four studied sections (S_1-S_4) along fibre length. The radial intensity distributions of the (110) Bragg reflection for polypropylene fibre (PP, Fibre 1) and polypropylene fibre with erucamide (PP + ER, Fibre 1) are shown in Figure 3 in the main text.

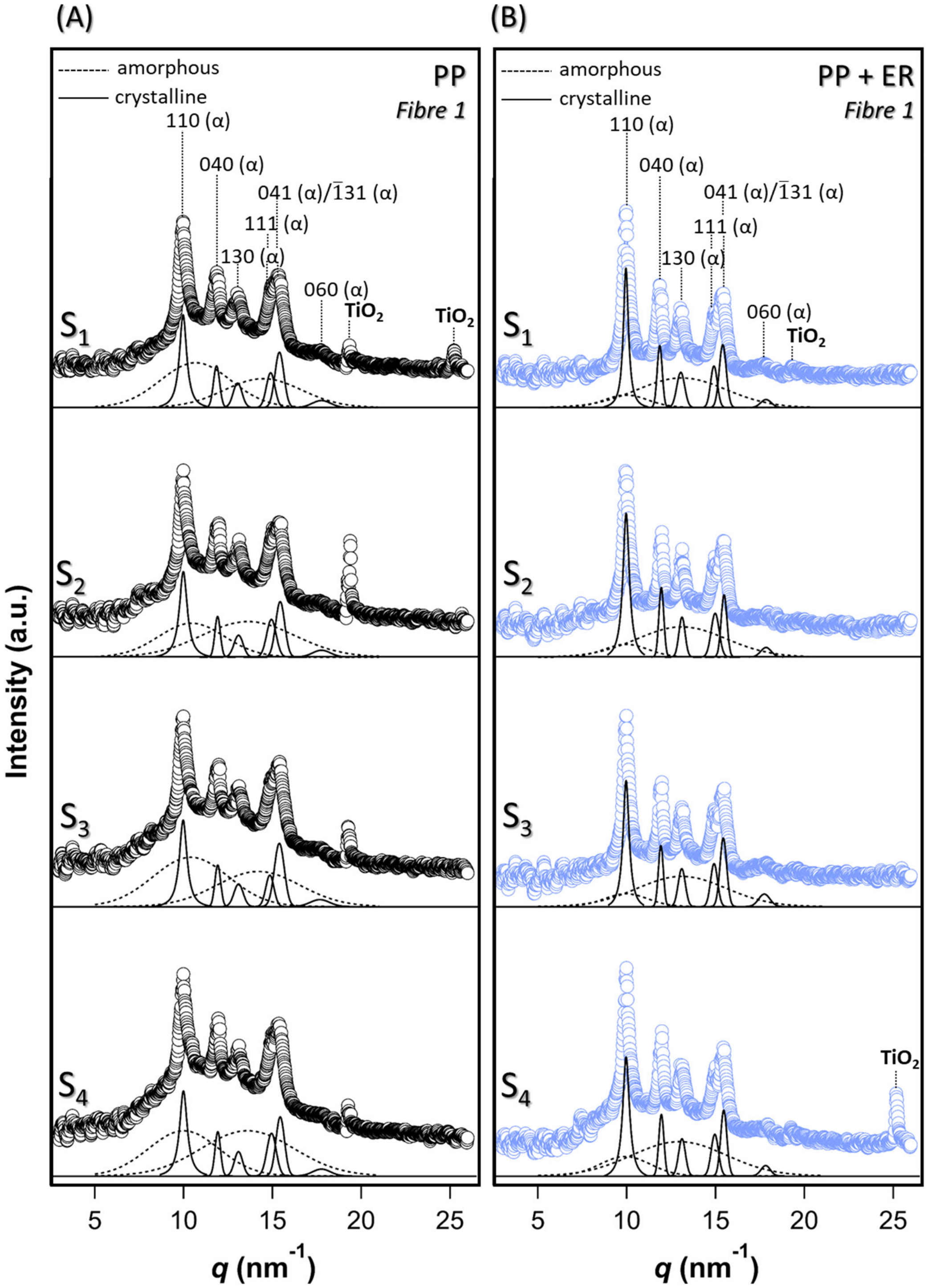

Figure S9 Experimental XRD curves obtained at four sections (S_1-S_4) along the fibre length for a polypropylene (PP) (A) and a polypropylene fibre with erucamide (PP + ER) (B). The dashed lines indicate the Gaussian fit for the amorphous halo (*i.e.* the amorphous fraction) and the solid lines the Gaussian and Voigt fit for the Bragg peaks (i.e. the crystalline fraction).

Figure S10 shows the experimental XRD curves obtained for replicates (Fibre 2) of PP and PP + ER fibre at four studied sections (S_1-S_4) along fibre length (Fibre 2). Figure S11 shows the representative radial intensity distribution of (110) Bragg reflection obtained for polypropylene fibre (PP, Fibre 2) and polypropylene fibre with erucamide (PP + ER, Fibre 2). Fitting of the data allowed to estimate the crystallite size and the degree of crystallinity. The fitting results are listed in main manuscript.

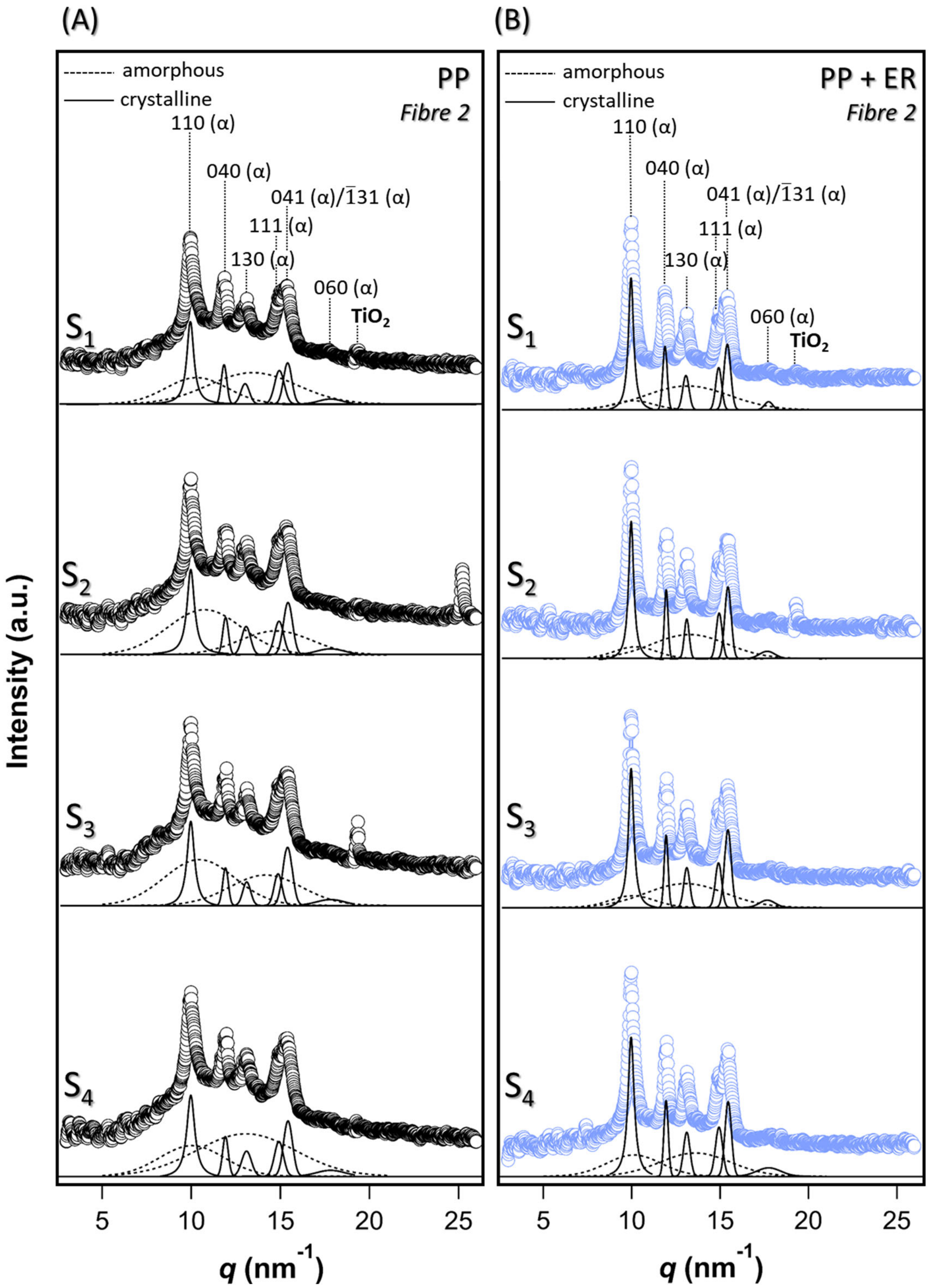

Figure S10 Experimental XRD curves obtained at four sections (S_1-S_4) along fibre length for single polypropylene fibre (PP) and polypropylene fibre with erucamide masterbatch (PP + ER). The dashed lines indicate the Gaussian fits for the amorphous halo (amorphous fraction) and the solid lines indicate Gaussian and Voigt fits for Bragg peaks (crystalline fraction).

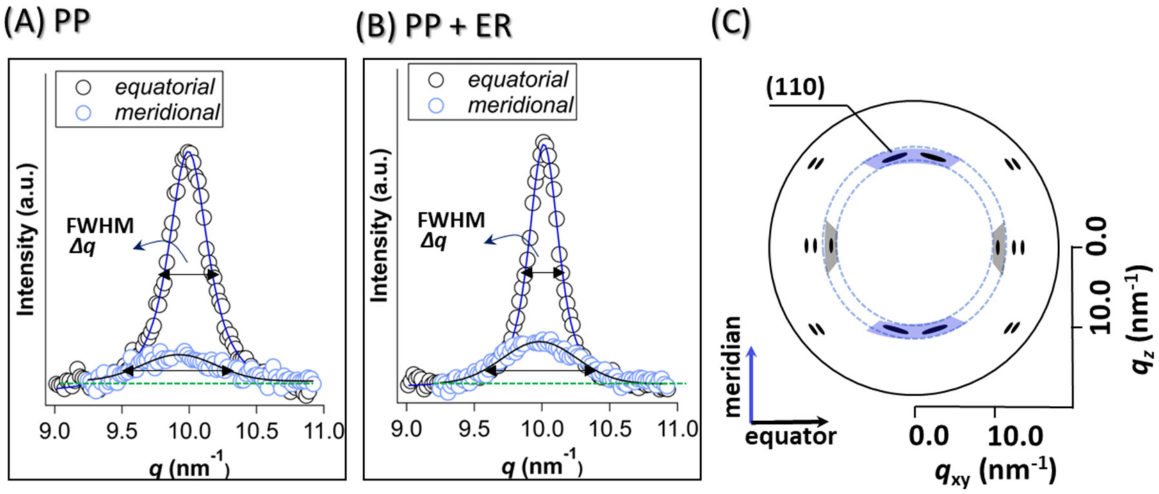

Figure S11 Representative radial intensity distributions of equatorial and near-meridional (110) reflections obtained for: (A) polypropylene fibre (PP, Fibre 2) and (B) polypropylene fibre with erucamide (PP + ER, Fibre 2); (C) Schematic representation of the 2D XRD pattern, with the radial region corresponding to equatorial reflections in grey and the radial region corresponding to near-meridional reflections in blue.

SI.10 Crystallite size and degree of crystallinity across fibre width

Table S5 lists the degree of crystallinity (D_c) calculated for PP and PP + ER for two samples per fibre type at three discrete positions denoted as A_1 (left edge), A_2 (core), A_3 (right edge) along S_1 across the fibre width (*cf.* Figure 4C in the main text).

Table S5 Degree of crystallinity (D_c) calculated using Equation S8 for polypropylene (PP) and polypropylene with erucamide (PP + ER) fibre at three discrete positions denoted as A_1 (left edge), A_2 (core), A_3 (right edge) along S_1 across the fibre width. The errors for D_c were calculated *via* the error propagation method based on the uncertainties in the integrated intensities under fitted Bragg peaks (A_c) and the amorphous halo (A_a).

Section:	Degree of crystallinity D_c (%)	
	PP	PP + ER

	A_1	A_2	A_3	A_1	A_2	A_3
**Fibre 1**	29 ± 3	34 ± 2	28 ± 3	44 ± 4	44 ± 2	44 ± 3
**Fibre 2**	31 ± 3	33 ± 2	29 ± 3	50 ± 3	50 ± 2	50 ± 4

Figure S12 shows the experimental XRD curves obtained for replicates (Fibre 2) of PP and PP + ER fibre at three studied sections (A_1-A_3) across fibre width. Fitting of the data allowed to estimated the crystallite size and the degree of crystallinity. The fitting results are listed in the main manuscript. Figure S14 shows the representative radial intensity distribution of (110) Bragg reflection obtained for polypropylene fibre (PP, Fibre 2) and polypropylene fibre with erucamide (PP + ER, Fibre 2), obtained at three areas along fibre cross sections (A_1-A_3). Fitting of the data allowed to estimated the crystallite size and the degree of crystallinity. The fitting results are listed in the main manuscript.

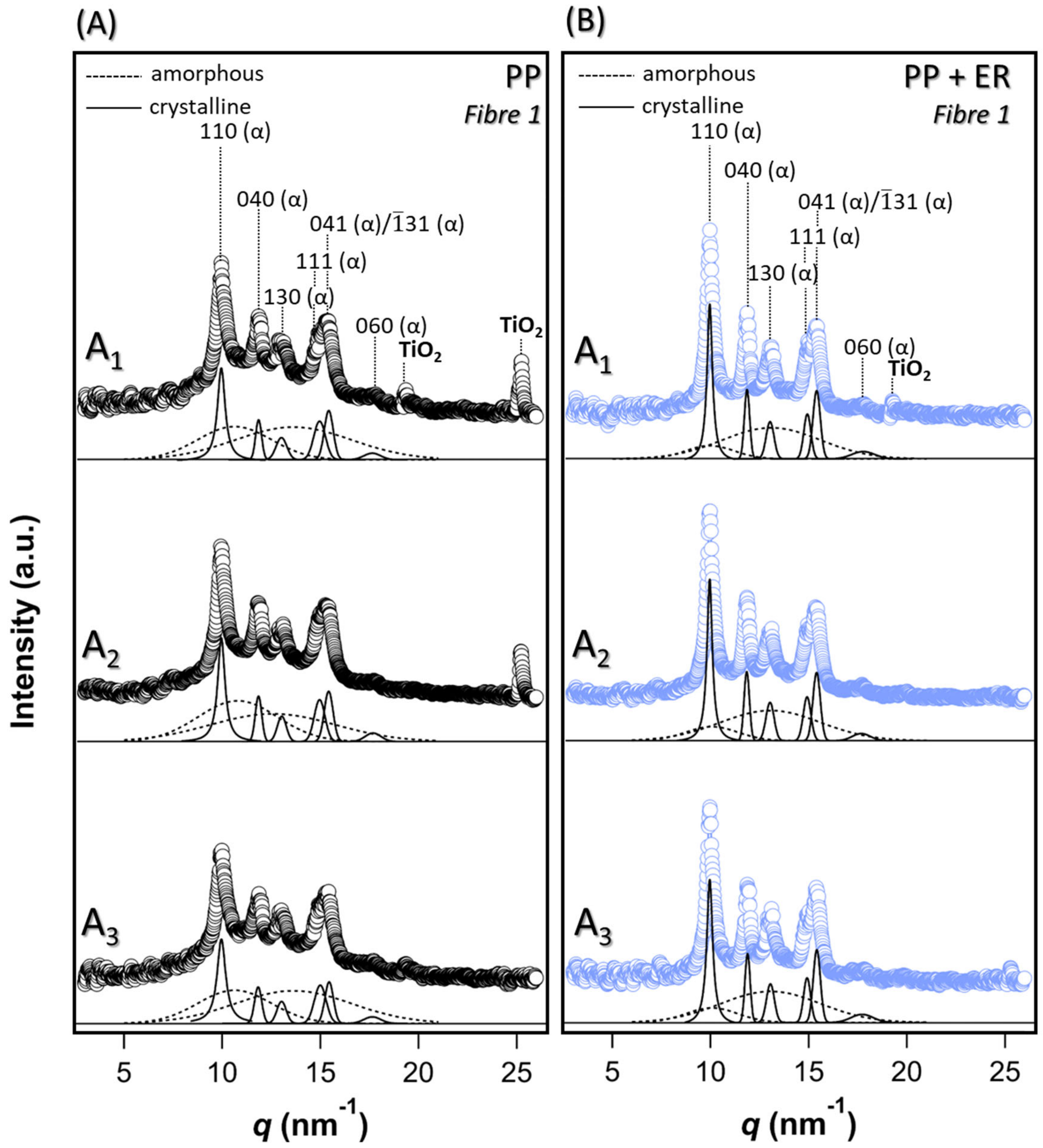

Figure S12 Experimental XRD curves along with the decomposed Gaussian and Voigt fits for the amorphous region (dotted lines) and the Bragg peaks (solid lines), obtained for the three sections (A_1-A_3) across the fibre width for single polypropylene fibres (PP; A, left) and single polypropylene fibres with erucamide (PP + ER; B, right). The individual fits obtained for the meridional and equatorial contributions to the (110) Bragg reflection and further results for other fibre samples (Fibre 2) are shown in Supplementary Information SI.05.

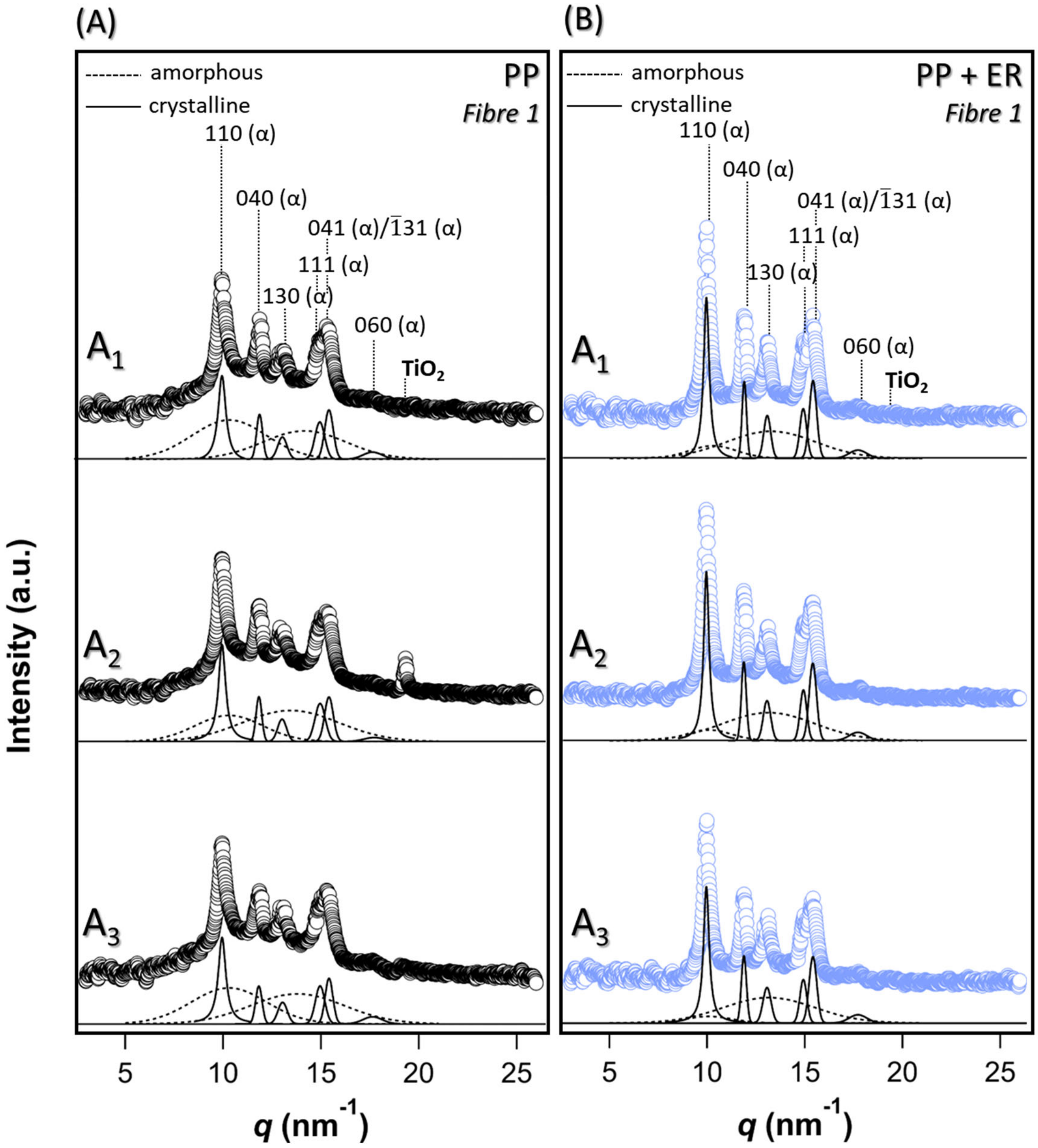

Figure S13 Experimental XRD curves obtained at three sections (A_1-A_3) across fibre width for single polypropylene fibres (PP) (left) and single polypropylene fibres with erucamide masterbatch (right).

Table S6 Fitted structural parameters for polypropylene fibre (PP) and polypropylene fibre with erucamide (PP + ER) measured at three sections (A_1-A_3) across the fibre width: the crystallite size (L_a) and the number of chains (m) ordered in the direction normal to the

direction of the plane (*hkl*) giving rise to the reflection. The fitted structural parameters corresponding to the equatorial (parent lamellae) and the meridional (daughter lamellae) contributions to (110) reflection are denoted as 110 *eq*. and 110 *m.*, respectively. The errors for L_a were calculated *via* error propagation using the uncertainties for FWHM (Δq). The errors for *m* calculated as a combination of uncertainties in *d* and L_a are all smaller than 1.

Sample		*hkl*	L_a (Å)			*m*		
			A_1	A_2	A_3	A_1	A_2	A_3
PP	Fibre 1	110 *eq.*	138 ± 10	163 ± 1	115 ± 1	22	26	18
		110 *m.*	72 ± 1	89 ± 1	95 ± 1	11	14	15
		040	175 ± 31	164 ± 24	146 ± 24	33	31	28
		130	98 ± 24	116 ± 26	113 ± 27	20	24	23
		111	90 ± 53	98 ± 49	89 ± 48	21	23	21
		041/$\bar{1}$31	117 ± 38	119 ± 37	123 ± 43	29	29	30
		060	63 ± 40	67 ± 52	63 ± 40	18	19	18
	Fibre 2	110 *eq.*	170 ± 1	186 ± 1	172 ± 7	27	29	27
		110 *m.*	115 ± 2	102 ± 1	88 ± 1	18	16	14
		040	175 ± 28	181 ± 26	161 ± 28	33	34	30
		130	112 ± 29	118 ± 28	112 ± 19	23	24	23
		111	108 ± 58	102 ± 52	111 ± 17	26	24	26
		041/$\bar{1}$31	122 ± 42	121 ± 41	136 ± 14	30	30	33
		060	55 ± 6	50 ± 33	55 ± 6	15	14	15
PP + ER	Fibre 1	110 *eq.*	245 ± 9	248 ± 5	261 ± 2	39	39	41
		110 *m.*	73 ± 1	95 ± 1	104 ± 1	12	15	17
		040	197 ± 24	196 ± 24	202 ± 28	37	37	38
		130	133 ± 26	130 ± 23	133 ± 27	28	27	28
		111	147 ± 47	137 ± 45	150 ± 48	35	33	36
		041/$\bar{1}$31	143 ± 30	142 ± 29	146 ± 29	35	35	36
		060	46 ± 27	60 ± 39	47 ± 5	13	17	13
	Fibre 2	110 *eq.*	234 ± 6	246 ± 3	245 ± 6	37	39	39
		110 *m.*	111 ± 1	107 ± 1	100 ± 1	18	17	16
		040	233 ± 31	214 ± 26	205 ± 27	44	40	39
		130	150 ± 29	139 ± 27	126 ± 26	31	29	26
		111	153 ± 42	155 ± 43	151 ± 36	36	37	36
		041/$\bar{1}$31	142 ± 25	140 ± 26	137 ± 34	35	34	34
		060	60 ± 31	60 ± 31	60 ± 31	17	17	17

Table S7 Relative content of the parent and daughter lamellae based on the relative peak intensity of the equatorial and meridional contributions to the (110) Bragg reflection, for PP

fibre and PP fibre with 1.5 wt% erucamide (PP + ER) at three sections (A_1-A_3) across the fibre width.

**Sample**	**Lamellae**	**Relative content (%)**					
		Fibre 1			***Fibre 2***		
		**A_1**	**A_2**	**A_3**	**A_1**	**A_2**	**A_3**
**PP**	***parent***	88	89	89	90	89	87
	daughter	12	11	11	10	11	13
**PP + ER**	***parent***	84	82	84	85	83	86
	daughter	16	18	16	15	17	14

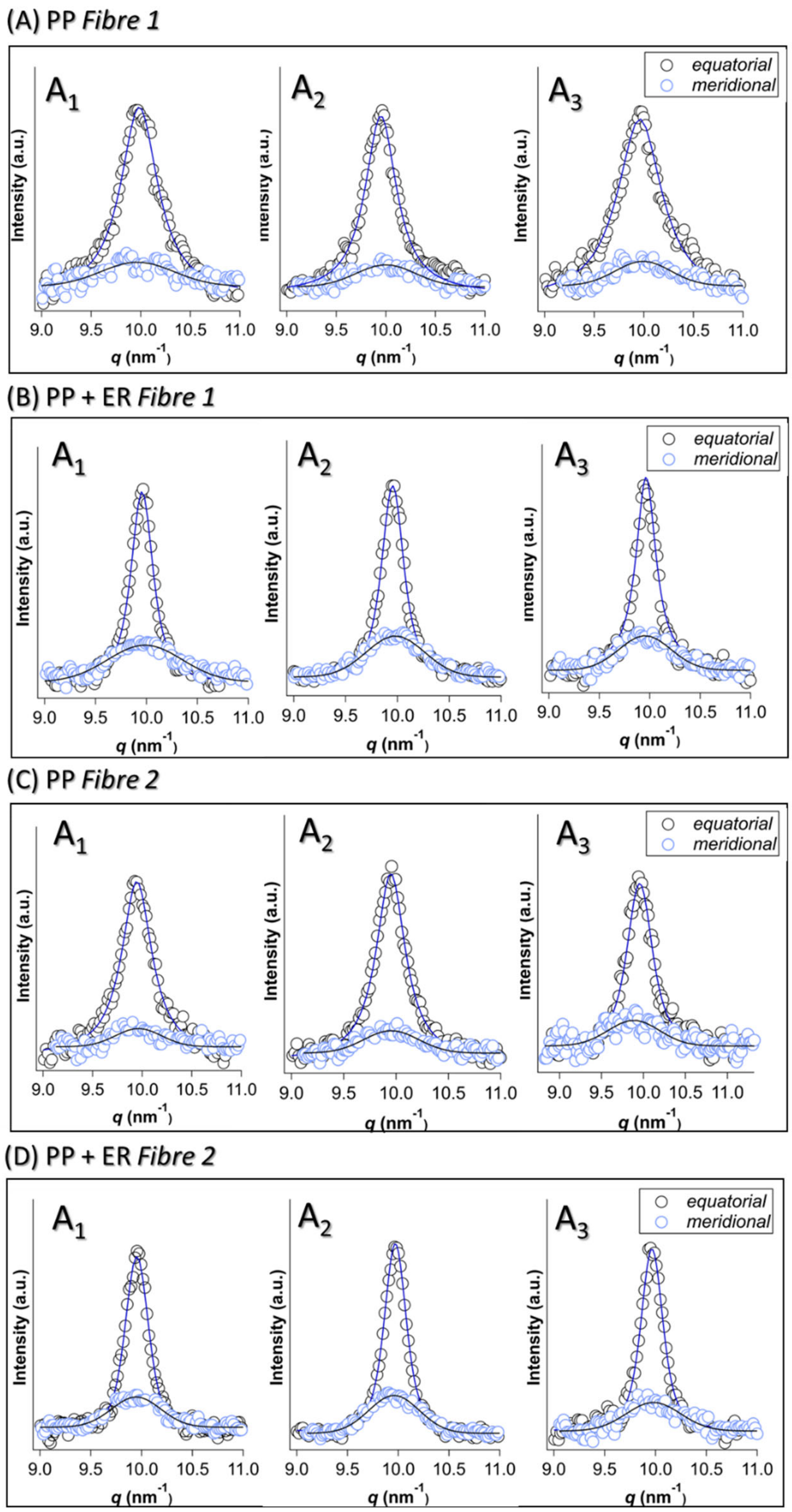

Figure S14 Radial intensity distributions of equatorial and near-meridional (110) reflections obtained at three sections across fibre width (A_1-A_3) for: (A) and (C) polypropylene fibre (PP) and (B) and (D) polypropylene fibre with erucamide (PP + ER).

SI.11 Differential Scanning Calorimetry

Thermal analysis was conducted at Procter & Gamble Technology (Beijing) Co., Ltd., using differential scanning calorimetry (DSC Q2000, TA Instruments) in the temperature range 20-200 °C and the heating rate of 10°C min^{-1}. The instrument was calibrated for cell resistance and capacitance differences, heat flow constant, and temperature. The cell resistance and capacitance calibration compensated for the subtle difference in thermal resistance and capacitance between the reference and sample platforms in the DSC sensor. The calibration was based on two experiments – one run with an empty cell, and the second run with equal weight sapphire disks on the sample and reference platforms. The cell constant is a calibration factor used to adjust for subtle differences in the calorimetric response of a DSC cell; whilst the temperature calibration ensures that the sample thermocouple reading is correct under the experimental condition chosen. Both of these calibrations were performed on the melting peak of a high-purity standard metal (i.e. Indium) used for our instrument. The first heating cycle was followed by cooling and next second heating cycle, all attained under the same controlled rate. Two PP samples and two PP + ER samples were studied. The results are shown in Figure S15 and point to the presence of erucamide affecting the fibre melting temperature, yielding at a higher value for PP + ER (T_m = 160.7 ± 0.1 °C) than that for pure PP (T_m = 149.1 ± 0.2 °C).

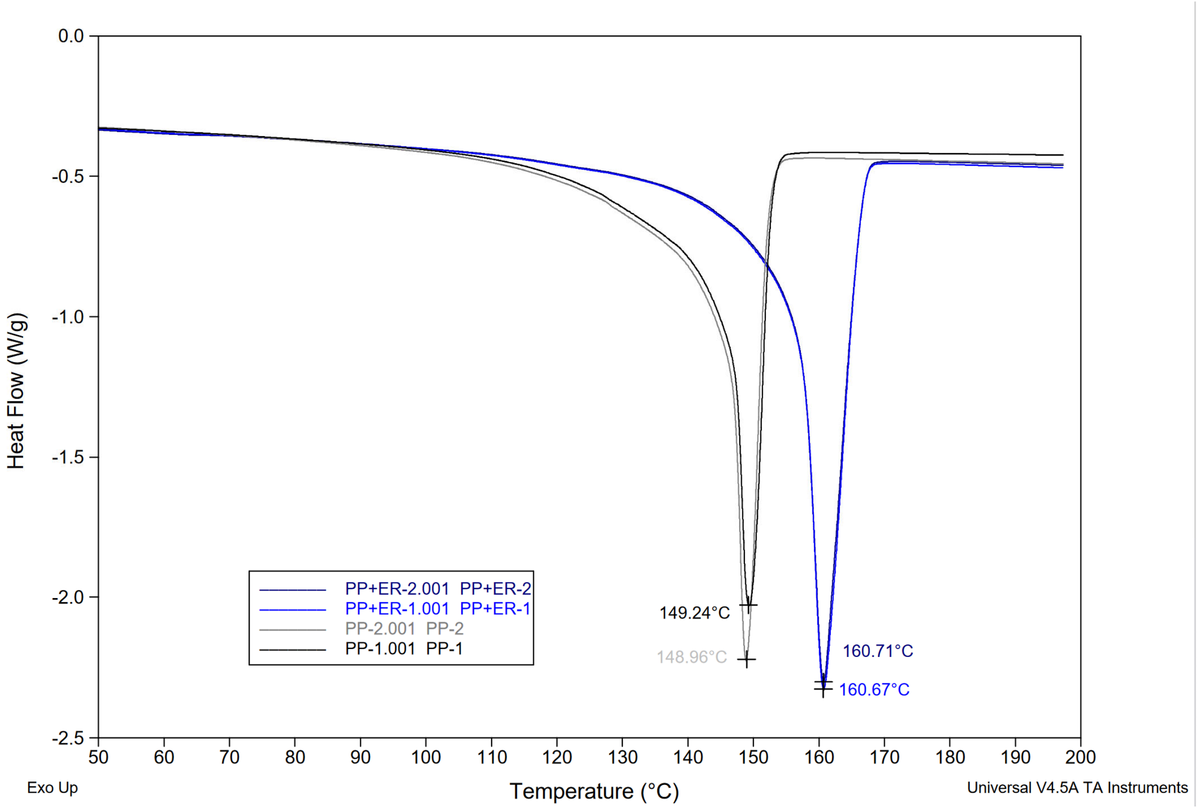

Figure S15 The DSC curves obtained for PP and two PP + ER samples (2 repeats each). The curves correspond to second heating cycle, after removing the thermal history and a re-crystallization process under the same controlled rate.

SI.12. Mechanical properties

Table S8 Mechanical parameters obtained for ten specimens per sample for PP and PP + ER fibres: fibre diameter (d_f), engineering tensile strength (σ), engineering elongation at break (ε), true tensile strength (σ_t), true elongation at break (ε_t), Young's modulus (E), yield strength (σ_y), and toughness (T). The errors for the average values were calculated as a standard deviation from 10 fibres per sample.

	Sample no.	d_f (µm)	σ (MPa)	ε (%)	σ_t (MPa)	ε_t (%)	E (GPa)	σ_y (MPa)	T (Jm^{-3})
PP	**1**	12.5	311	73	536	55	1.2	159	134
	**2**	13.8	329	78	585	58	1.0	195	156
	**3**	14.3	304	71	521	54	1.2	170	139
	**4**	13.0	364	75	636	56	1.3	214	177
	**5**	12.7	336	63	546	49	1.7	215	139

	**6**	14.4	344	79	615	58	1.6	198	180
	**7**	12.2	349	63	569	49	1.6	219	142
	**8**	13.7	348	73	603	55	1.5	196	162
	**9**	12.3	370	64	593	49	1.4	230	150
	**10**	11.1	385	69	651	52	1.7	213	154
	**Average**	**13.0**	**344**	**71**	**586**	**53**	**1.5**	**201**	**153**
	**Error**	**1.1**	**25**	**6**	**43**	**3**	**0.3**	**22**	**16**
**PP + ER**	**1**	13.4	189	163	496	97	1.4	84	188
	**2**	13.4	180	165	478	97	1.1	76	179
	**3**	13.4	195	166	518	98	1.3	77	203
	**4**	12.2	197	148	487	91	1.3	75	180
	**5**	11.5	191	157	458	95	0.9	94	183
	**6**	13.4	192	164	508	97	1.5	80	189
	**7**	12.2	186	167	496	98	1.1	76	181
	**8**	12.8	195	164	512	97	1.2	84	197
	**9**	12.4	195	162	511	96	1.2	81	189
	**10**	12.9	200	162	525	96	1.1	76	196
	**Average**	**12.8**	**192**	**162**	**499**	**96**	**1.2**	**80**	**188**
	**Error**	**0.7**	**6**	**6**	**20**	**2**	**0.2**	**6**	**8**

SI.12. True engineering stress vs strain curves for PP and PP+ER si

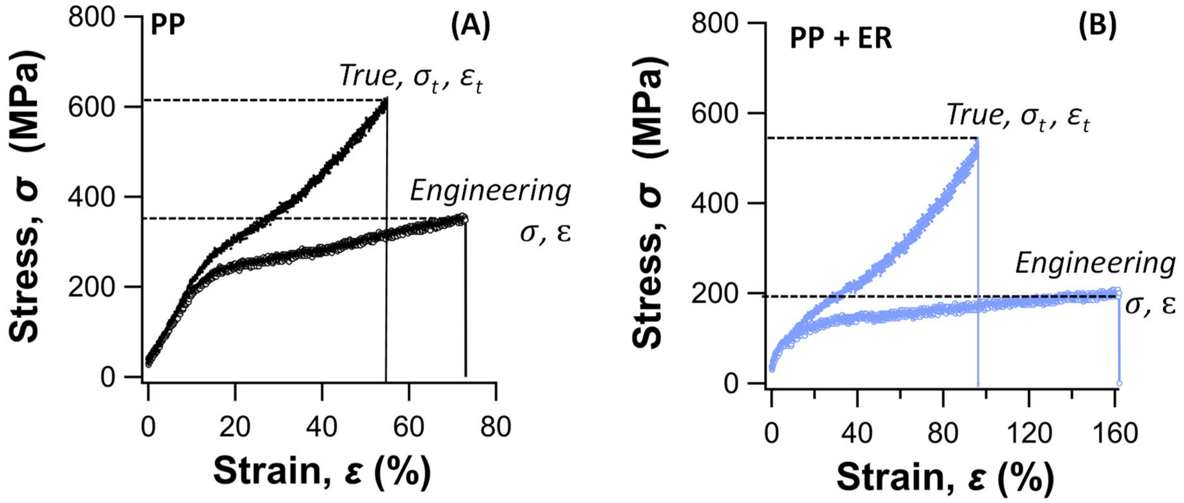

Figure S16 Comparison between true and engineering stress *vs* strain (σ *vs* ε) curves for both PP (A) and PP + ER (B). The slope of the elastic region gives the Young's modulus (*E*) associated with fibre stiffness. The maximum stress and the elongation (*i.e.* in percentage of the fibre starting length) before fracture are defined as the engineering tensile strength (σ) and the

fracture strain (*i.e*. elongation at break, ε), respectively, and they are calculated based on the original cross-section and gauge length. It is commonly accepted that the displacement of fibrils during plastic deformation upon stretching results in a reduction in the total number of fibrils per cross-section (*i.e.* necking). To account for these changes, the true stress (σ_t) and true strain (ε_t) can be calculated based on the instantaneous cross-section area and fibre length *[22]*.

References

1. Stribeck, N., *On the determination of fiber tilt angles in fiber diffraction.* Acta crystallographica. Section A, Foundations of crystallography, 2009. **65**: p. 46-7.
2. Quinn, P.D., et al., *The Hard X-ray Nanoprobe beamline at Diamond Light Source.* 2021. **28**(3).
3. Parker, J.E., et al., *A cell design for correlative hard X-ray nanoprobe and electron microscopy studies of catalysts under in situ conditions.* Journal of Synchrotron Radiation, 2022. **29**: p. 431-438.
4. Filik, J., et al., *Processing two-dimensional X-ray diffraction and small-angle scattering data in DAWN 2.* Journal of Applied Crystallography, 2017. **50**: p. 959-966.
5. Heiney, P.A., (2005), IUCr Commission on Powder Diffraction Newsletter, (No. 32): p. 9–11.
6. Fraser, R., et al., *Digital processing of fibre diffraction patterns.* Journal of Applied Crystallography, 1976. **9**(2): p. 81-94.
7. Kahn, R., et al., *Macromolecular crystallography with synchrotron radiation: photographic data collection and polarization correction.* Journal of Applied Crystallography, 1982. **15**(3): p. 330-337.
8. Eilers, P.H. and H.F. Boelens, *Baseline correction with asymmetric least squares smoothing.* Leiden University Medical Centre Report, 2005. **1**(1): p. 5.
9. Patterson, A.L., *The Scherrer Formula for X-Ray Particle Size Determination.* Physical Review, 1939. **56**(10): p. 978-982.
10. Dane, T.G., et al., *Influence of solvent polarity on the structure of drop-cast electroactive tetra(aniline)-surfactant thin films.* Phys Chem Chem Phys, 2016. **18**(35): p. 24498-505.
11. Dane, T.G., et al., *Structured oligo(aniline) nanofilms via ionic self-assembly.* Soft Matter, 2012. **8**(10): p. 2824-2832.
12. Dane, T.G., et al., *Oligo(aniline) nanofilms: from molecular architecture to microstructure.* Soft Matter, 2013. **9**(44): p. 10501-10511.
13. Fox, L., J., Matthews, L., Stockdale, H., Pichai, S., Snow, T., Richardson, R. M., Briscoe, W. H., *Structure Changes in Lipid Mesophases due to Intercalation of Dendritic Polymer Nanoparticles: Swollen Lamellae, Suppressed Curvature, and Augmented Structural Disorder.* Acta Biomater, 2019. **104**: p. 198-209.

14. Fox, L., J., Slastanova, A., Taylor, N., Wlodek, M., Bikondoa, O., Richardson, R. M., Briscoe, W. H., *Interactions between PAMAM dendrimers and DOPC lipid multilayers: Membrane thinning and structural disorder.* Biochimica et Biophysica Acta (BBA) - General Subjects, 2020. **1865**(4): p. 129542.
15. Bulpett, J.M., et al., *Interactions of nanoparticles with purple membrane films.* J Mater Chem, 2012. **22**(31): p. 15635-15643.
16. Bulpett, J.M., et al., *Hydrophobic nanoparticles promote lamellar to inverted hexagonal transition in phospholipid mesophases.* Soft Matter, 2015. **11**(45): p. 8789-800.
17. Kaus, N.H.M., et al., *In situ X-ray reflectivity studies of molecular and molecular-cluster intercalation within purple membrane films.* J Mater Chem C, 2014. **2**(27): p. 5447-5452.
18. Hammond, C., (2009), *The basics of crystallography and diffraction*. Third edition. ed, ed. C. Hammond. Oxford University Press: Oxford Science Publications. International Union of Crystallography p. 383.
19. Karacan, I. and H. Benli, *An X-ray Diffraction study for isotactic polypropylene fibers produced with take-up speeds of 2500-4250 m/min.* Tekstil ve Konfeksiyon 2011. **21(3)**: p. 201-209.
20. Dia-stron. *Automated dimensional and tensile testing of carbon fibres (Accessed 15th May 2022)*. Available from: https://www.diastron.com/resources/carbon-fibre-application-note/.
21. Gubała, D., et al., *Multiscale characterisation of single synthetic fibres: Surface morphology and nanomechanical properties.* Journal of Colloid and Interface Science, 2020. **571**: p. 398-411.
22. Peterlin, A., *Plastic deformation of polymers with fibrous structure.* Colloid Polymer Science, 1975. **253**(10): p. 809-823.
